# Measurement of event-shape observables in $Z \rightarrow \ell ^{+} \ell ^{-}$ events in *pp* collisions at $\sqrt{s}=7$ ${\mathrm{TeV}}$ with the ATLAS detector at the LHC

**DOI:** 10.1140/epjc/s10052-016-4176-8

**Published:** 2016-07-06

**Authors:** Atlas Collaboration, G. Aad, B. Abbott, J. Abdallah, O. Abdinov, B. Abeloos, R. Aben, M. Abolins, O. S. AbouZeid, N. L. Abraham, H. Abramowicz, H. Abreu, R. Abreu, Y. Abulaiti, B. S. Acharya, L. Adamczyk, D. L. Adams, J. Adelman, S. Adomeit, T. Adye, A. A. Affolder, T. Agatonovic-Jovin, J. Agricola, J. A. Aguilar-Saavedra, S. P. Ahlen, F. Ahmadov, G. Aielli, H. Akerstedt, T. P. A. Åkesson, A. V. Akimov, G. L. Alberghi, J. Albert, S. Albrand, M. J. Alconada Verzini, M. Aleksa, I. N. Aleksandrov, C. Alexa, G. Alexander, T. Alexopoulos, M. Alhroob, M. Aliev, G. Alimonti, J. Alison, S. P. Alkire, B. M. M. Allbrooke, B. W. Allen, P. P. Allport, A. Aloisio, A. Alonso, F. Alonso, C. Alpigiani, B. Alvarez Gonzalez, D. Álvarez Piqueras, M. G. Alviggi, B. T. Amadio, K. Amako, Y. Amaral Coutinho, C. Amelung, D. Amidei, S. P. Amor Dos Santos, A. Amorim, S. Amoroso, N. Amram, G. Amundsen, C. Anastopoulos, L. S. Ancu, N. Andari, T. Andeen, C. F. Anders, G. Anders, J. K. Anders, K. J. Anderson, A. Andreazza, V. Andrei, S. Angelidakis, I. Angelozzi, P. Anger, A. Angerami, F. Anghinolfi, A. V. Anisenkov, N. Anjos, A. Annovi, M. Antonelli, A. Antonov, J. Antos, F. Anulli, M. Aoki, L. Aperio Bella, G. Arabidze, Y. Arai, J. P. Araque, A. T. H. Arce, F. A. Arduh, J.-F. Arguin, S. Argyropoulos, M. Arik, A. J. Armbruster, L. J. Armitage, O. Arnaez, H. Arnold, M. Arratia, O. Arslan, A. Artamonov, G. Artoni, S. Artz, S. Asai, N. Asbah, A. Ashkenazi, B. Åsman, L. Asquith, K. Assamagan, R. Astalos, M. Atkinson, N. B. Atlay, K. Augsten, G. Avolio, B. Axen, M. K. Ayoub, G. Azuelos, M. A. Baak, A. E. Baas, M. J. Baca, H. Bachacou, K. Bachas, M. Backes, M. Backhaus, P. Bagiacchi, P. Bagnaia, Y. Bai, J. T. Baines, O. K. Baker, E. M. Baldin, P. Balek, T. Balestri, F. Balli, W. K. Balunas, E. Banas, Sw. Banerjee, A. A. E. Bannoura, L. Barak, E. L. Barberio, D. Barberis, M. Barbero, T. Barillari, M. Barisonzi, T. Barklow, N. Barlow, S. L. Barnes, B. M. Barnett, R. M. Barnett, Z. Barnovska, A. Baroncelli, G. Barone, A. J. Barr, L. Barranco Navarro, F. Barreiro, J. Barreiro Guimarães da Costa, R. Bartoldus, A. E. Barton, P. Bartos, A. Basalaev, A. Bassalat, A. Basye, R. L. Bates, S. J. Batista, J. R. Batley, M. Battaglia, M. Bauce, F. Bauer, H. S. Bawa, J. B. Beacham, M. D. Beattie, T. Beau, P. H. Beauchemin, P. Bechtle, H. P. Beck, K. Becker, M. Becker, M. Beckingham, C. Becot, A. J. Beddall, A. Beddall, V. A. Bednyakov, M. Bedognetti, C. P. Bee, L. J. Beemster, T. A. Beermann, M. Begel, J. K. Behr, C. Belanger-Champagne, A. S. Bell, W. H. Bell, G. Bella, L. Bellagamba, A. Bellerive, M. Bellomo, K. Belotskiy, O. Beltramello, N. L. Belyaev, O. Benary, D. Benchekroun, M. Bender, K. Bendtz, N. Benekos, Y. Benhammou, E. Benhar Noccioli, J. Benitez, J. A. Benitez Garcia, D. P. Benjamin, J. R. Bensinger, S. Bentvelsen, L. Beresford, M. Beretta, D. Berge, E. Bergeaas Kuutmann, N. Berger, F. Berghaus, J. Beringer, S. Berlendis, N. R. Bernard, C. Bernius, F. U. Bernlochner, T. Berry, P. Berta, C. Bertella, G. Bertoli, F. Bertolucci, I. A. Bertram, C. Bertsche, D. Bertsche, G. J. Besjes, O. Bessidskaia Bylund, M. Bessner, N. Besson, C. Betancourt, S. Bethke, A. J. Bevan, W. Bhimji, R. M. Bianchi, L. Bianchini, M. Bianco, O. Biebel, D. Biedermann, R. Bielski, N. V. Biesuz, M. Biglietti, J. Bilbao De Mendizabal, H. Bilokon, M. Bindi, S. Binet, A. Bingul, C. Bini, S. Biondi, D. M. Bjergaard, C. W. Black, J. E. Black, K. M. Black, D. Blackburn, R. E. Blair, J.-B. Blanchard, J. E. Blanco, T. Blazek, I. Bloch, C. Blocker, W. Blum, U. Blumenschein, S. Blunier, G. J. Bobbink, V. S. Bobrovnikov, S. S. Bocchetta, A. Bocci, C. Bock, M. Boehler, D. Boerner, J. A. Bogaerts, D. Bogavac, A. G. Bogdanchikov, C. Bohm, V. Boisvert, T. Bold, V. Boldea, A. S. Boldyrev, M. Bomben, M. Bona, M. Boonekamp, A. Borisov, G. Borissov, J. Bortfeldt, D. Bortoletto, V. Bortolotto, K. Bos, D. Boscherini, M. Bosman, J. D. Bossio Sola, J. Boudreau, J. Bouffard, E. V. Bouhova-Thacker, D. Boumediene, C. Bourdarios, S. K. Boutle, A. Boveia, J. Boyd, I. R. Boyko, J. Bracinik, A. Brandt, G. Brandt, O. Brandt, U. Bratzler, B. Brau, J. E. Brau, H. M. Braun, W. D. Breaden Madden, K. Brendlinger, A. J. Brennan, L. Brenner, R. Brenner, S. Bressler, T. M. Bristow, D. Britton, D. Britzger, F. M. Brochu, I. Brock, R. Brock, G. Brooijmans, T. Brooks, W. K. Brooks, J. Brosamer, E. Brost, J. H. Broughton, P. A. Bruckman de Renstrom, D. Bruncko, R. Bruneliere, A. Bruni, G. Bruni, B. H. Brunt, M. Bruschi, N. Bruscino, P. Bryant, L. Bryngemark, T. Buanes, Q. Buat, P. Buchholz, A. G. Buckley, I. A. Budagov, F. Buehrer, M. K. Bugge, O. Bulekov, D. Bullock, H. Burckhart, S. Burdin, C. D. Burgard, B. Burghgrave, K. Burka, S. Burke, I. Burmeister, E. Busato, D. Büscher, V. Büscher, P. Bussey, J. M. Butler, A. I. Butt, C. M. Buttar, J. M. Butterworth, P. Butti, W. Buttinger, A. Buzatu, A. R. Buzykaev, S. Cabrera Urbán, D. Caforio, V. M. Cairo, O. Cakir, N. Calace, P. Calafiura, A. Calandri, G. Calderini, P. Calfayan, L. P. Caloba, D. Calvet, S. Calvet, T. P. Calvet, R. Camacho Toro, S. Camarda, P. Camarri, D. Cameron, R. Caminal Armadans, C. Camincher, S. Campana, M. Campanelli, A. Campoverde, V. Canale, A. Canepa, M. Cano Bret, J. Cantero, R. Cantrill, T. Cao, M. D. M. Capeans Garrido, I. Caprini, M. Caprini, M. Capua, R. Caputo, R. M. Carbone, R. Cardarelli, F. Cardillo, T. Carli, G. Carlino, L. Carminati, S. Caron, E. Carquin, G. D. Carrillo-Montoya, J. R. Carter, J. Carvalho, D. Casadei, M. P. Casado, M. Casolino, D. W. Casper, E. Castaneda-Miranda, A. Castelli, V. Castillo Gimenez, N. F. Castro, A. Catinaccio, J. R. Catmore, A. Cattai, J. Caudron, V. Cavaliere, E. Cavallaro, D. Cavalli, M. Cavalli-Sforza, V. Cavasinni, F. Ceradini, L. Cerda Alberich, B. C. Cerio, A. S. Cerqueira, A. Cerri, L. Cerrito, F. Cerutti, M. Cerv, A. Cervelli, S. A. Cetin, A. Chafaq, D. Chakraborty, I. Chalupkova, S. K. Chan, Y. L. Chan, P. Chang, J. D. Chapman, D. G. Charlton, A. Chatterjee, C. C. Chau, C. A. Chavez Barajas, S. Che, S. Cheatham, A. Chegwidden, S. Chekanov, S. V. Chekulaev, G. A. Chelkov, M. A. Chelstowska, C. Chen, H. Chen, K. Chen, S. Chen, S. Chen, X. Chen, Y. Chen, H. C. Cheng, H. J. Cheng, Y. Cheng, A. Cheplakov, E. Cheremushkina, R. Cherkaoui El Moursli, V. Chernyatin, E. Cheu, L. Chevalier, V. Chiarella, G. Chiarelli, G. Chiodini, A. S. Chisholm, A. Chitan, M. V. Chizhov, K. Choi, A. R. Chomont, S. Chouridou, B. K. B. Chow, V. Christodoulou, D. Chromek-Burckhart, J. Chudoba, A. J. Chuinard, J. J. Chwastowski, L. Chytka, G. Ciapetti, A. K. Ciftci, D. Cinca, V. Cindro, I. A. Cioara, A. Ciocio, F. Cirotto, Z. H. Citron, M. Ciubancan, A. Clark, B. L. Clark, M. R. Clark, P. J. Clark, R. N. Clarke, C. Clement, Y. Coadou, M. Cobal, A. Coccaro, J. Cochran, L. Coffey, L. Colasurdo, B. Cole, S. Cole, A. P. Colijn, J. Collot, T. Colombo, G. Compostella, P. Conde Muiño, E. Coniavitis, S. H. Connell, I. A. Connelly, V. Consorti, S. Constantinescu, C. Conta, G. Conti, F. Conventi, M. Cooke, B. D. Cooper, A. M. Cooper-Sarkar, T. Cornelissen, M. Corradi, F. Corriveau, A. Corso-Radu, A. Cortes-Gonzalez, G. Cortiana, G. Costa, M. J. Costa, D. Costanzo, G. Cottin, G. Cowan, B. E. Cox, K. Cranmer, S. J. Crawley, G. Cree, S. Crépé-Renaudin, F. Crescioli, W. A. Cribbs, M. Crispin Ortuzar, M. Cristinziani, V. Croft, G. Crosetti, T. Cuhadar Donszelmann, J. Cummings, M. Curatolo, J. Cúth, C. Cuthbert, H. Czirr, P. Czodrowski, S. D’Auria, M. D’Onofrio, M. J. Da Cunha Sargedas De Sousa, C. Da Via, W. Dabrowski, T. Dai, O. Dale, F. Dallaire, C. Dallapiccola, M. Dam, J. R. Dandoy, N. P. Dang, A. C. Daniells, N. S. Dann, M. Danninger, M. Dano Hoffmann, V. Dao, G. Darbo, S. Darmora, J. Dassoulas, A. Dattagupta, W. Davey, C. David, T. Davidek, M. Davies, P. Davison, Y. Davygora, E. Dawe, I. Dawson, R. K. Daya-Ishmukhametova, K. De, R. de Asmundis, A. De Benedetti, S. De Castro, S. De Cecco, N. De Groot, P. de Jong, H. De la Torre, F. De Lorenzi, D. De Pedis, A. De Salvo, U. De Sanctis, A. De Santo, J. B. De Vivie De Regie, W. J. Dearnaley, R. Debbe, C. Debenedetti, D. V. Dedovich, I. Deigaard, J. Del Peso, T. Del Prete, D. Delgove, F. Deliot, C. M. Delitzsch, M. Deliyergiyev, A. Dell’Acqua, L. Dell’Asta, M. Dell’Orso, M. Della Pietra, D. della Volpe, M. Delmastro, P. A. Delsart, C. Deluca, D. A. DeMarco, S. Demers, M. Demichev, A. Demilly, S. P. Denisov, D. Denysiuk, D. Derendarz, J. E. Derkaoui, F. Derue, P. Dervan, K. Desch, C. Deterre, K. Dette, P. O. Deviveiros, A. Dewhurst, S. Dhaliwal, A. Di Ciaccio, L. Di Ciaccio, W. K. Di Clemente, A. Di Domenico, C. Di Donato, A. Di Girolamo, B. Di Girolamo, A. Di Mattia, B. Di Micco, R. Di Nardo, A. Di Simone, R. Di Sipio, D. Di Valentino, C. Diaconu, M. Diamond, F. A. Dias, M. A. Diaz, E. B. Diehl, J. Dietrich, S. Diglio, A. Dimitrievska, J. Dingfelder, P. Dita, S. Dita, F. Dittus, F. Djama, T. Djobava, J. I. Djuvsland, M. A. B. do Vale, D. Dobos, M. Dobre, C. Doglioni, T. Dohmae, J. Dolejsi, Z. Dolezal, B. A. Dolgoshein, M. Donadelli, S. Donati, P. Dondero, J. Donini, J. Dopke, A. Doria, M. T. Dova, A. T. Doyle, E. Drechsler, M. Dris, Y. Du, J. Duarte-Campderros, E. Duchovni, G. Duckeck, O. A. Ducu, D. Duda, A. Dudarev, L. Duflot, L. Duguid, M. Dührssen, M. Dunford, H. Duran Yildiz, M. Düren, A. Durglishvili, D. Duschinger, B. Dutta, M. Dyndal, C. Eckardt, K. M. Ecker, R. C. Edgar, W. Edson, N. C. Edwards, T. Eifert, G. Eigen, K. Einsweiler, T. Ekelof, M. El Kacimi, V. Ellajosyula, M. Ellert, S. Elles, F. Ellinghaus, A. A. Elliot, N. Ellis, J. Elmsheuser, M. Elsing, D. Emeliyanov, Y. Enari, O. C. Endner, M. Endo, J. S. Ennis, J. Erdmann, A. Ereditato, G. Ernis, J. Ernst, M. Ernst, S. Errede, E. Ertel, M. Escalier, H. Esch, C. Escobar, B. Esposito, A. I. Etienvre, E. Etzion, H. Evans, A. Ezhilov, F. Fabbri, L. Fabbri, G. Facini, R. M. Fakhrutdinov, S. Falciano, R. J. Falla, J. Faltova, Y. Fang, M. Fanti, A. Farbin, A. Farilla, C. Farina, T. Farooque, S. Farrell, S. M. Farrington, P. Farthouat, F. Fassi, P. Fassnacht, D. Fassouliotis, M. Faucci Giannelli, A. Favareto, W. J. Fawcett, L. Fayard, O. L. Fedin, W. Fedorko, S. Feigl, L. Feligioni, C. Feng, E. J. Feng, H. Feng, A. B. Fenyuk, L. Feremenga, P. Fernandez Martinez, S. Fernandez Perez, J. Ferrando, A. Ferrari, P. Ferrari, R. Ferrari, D. E. Ferreira de Lima, A. Ferrer, D. Ferrere, C. Ferretti, A. Ferretto Parodi, F. Fiedler, A. Filipčič, M. Filipuzzi, F. Filthaut, M. Fincke-Keeler, K. D. Finelli, M. C. N. Fiolhais, L. Fiorini, A. Firan, A. Fischer, C. Fischer, J. Fischer, W. C. Fisher, N. Flaschel, I. Fleck, P. Fleischmann, G. T. Fletcher, G. Fletcher, R. R. M. Fletcher, T. Flick, A. Floderus, L. R. Flores Castillo, M. J. Flowerdew, G. T. Forcolin, A. Formica, A. Forti, A. G. Foster, D. Fournier, H. Fox, S. Fracchia, P. Francavilla, M. Franchini, D. Francis, L. Franconi, M. Franklin, M. Frate, M. Fraternali, D. Freeborn, S. M. Fressard-Batraneanu, F. Friedrich, D. Froidevaux, J. A. Frost, C. Fukunaga, E. Fullana Torregrosa, T. Fusayasu, J. Fuster, C. Gabaldon, O. Gabizon, A. Gabrielli, A. Gabrielli, G. P. Gach, S. Gadatsch, S. Gadomski, G. Gagliardi, L. G. Gagnon, P. Gagnon, C. Galea, B. Galhardo, E. J. Gallas, B. J. Gallop, P. Gallus, G. Galster, K. K. Gan, J. Gao, Y. Gao, Y. S. Gao, F. M. Garay Walls, C. García, J. E. García Navarro, M. Garcia-Sciveres, R. W. Gardner, N. Garelli, V. Garonne, A. Gascon Bravo, C. Gatti, A. Gaudiello, G. Gaudio, B. Gaur, L. Gauthier, I. L. Gavrilenko, C. Gay, G. Gaycken, E. N. Gazis, Z. Gecse, C. N. P. Gee, Ch. Geich-Gimbel, M. P. Geisler, C. Gemme, M. H. Genest, C. Geng, S. Gentile, S. George, D. Gerbaudo, A. Gershon, S. Ghasemi, H. Ghazlane, M. Ghneimat, B. Giacobbe, S. Giagu, P. Giannetti, B. Gibbard, S. M. Gibson, M. Gignac, M. Gilchriese, T. P. S. Gillam, D. Gillberg, G. Gilles, D. M. Gingrich, N. Giokaris, M. P. Giordani, F. M. Giorgi, F. M. Giorgi, P. F. Giraud, P. Giromini, D. Giugni, F. Giuli, C. Giuliani, M. Giulini, B. K. Gjelsten, S. Gkaitatzis, I. Gkialas, E. L. Gkougkousis, L. K. Gladilin, C. Glasman, J. Glatzer, P. C. F. Glaysher, A. Glazov, M. Goblirsch-Kolb, J. Godlewski, S. Goldfarb, T. Golling, D. Golubkov, A. Gomes, R. Gonçalo, J. Goncalves Pinto Firmino Da Costa, L. Gonella, A. Gongadze, S. González de la Hoz, G. Gonzalez Parra, S. Gonzalez-Sevilla, L. Goossens, P. A. Gorbounov, H. A. Gordon, I. Gorelov, B. Gorini, E. Gorini, A. Gorišek, E. Gornicki, A. T. Goshaw, C. Gössling, M. I. Gostkin, C. R. Goudet, D. Goujdami, A. G. Goussiou, N. Govender, E. Gozani, L. Graber, I. Grabowska-Bold, P. O. J. Gradin, P. Grafström, J. Gramling, E. Gramstad, S. Grancagnolo, V. Gratchev, H. M. Gray, E. Graziani, Z. D. Greenwood, C. Grefe, K. Gregersen, I. M. Gregor, P. Grenier, K. Grevtsov, J. Griffiths, A. A. Grillo, K. Grimm, S. Grinstein, Ph. Gris, J.-F. Grivaz, S. Groh, J. P. Grohs, E. Gross, J. Grosse-Knetter, G. C. Grossi, Z. J. Grout, L. Guan, W. Guan, J. Guenther, F. Guescini, D. Guest, O. Gueta, E. Guido, T. Guillemin, S. Guindon, U. Gul, C. Gumpert, J. Guo, Y. Guo, S. Gupta, G. Gustavino, P. Gutierrez, N. G. Gutierrez Ortiz, C. Gutschow, C. Guyot, C. Gwenlan, C. B. Gwilliam, A. Haas, C. Haber, H. K. Hadavand, N. Haddad, A. Hadef, P. Haefner, S. Hageböck, Z. Hajduk, H. Hakobyan, M. Haleem, J. Haley, D. Hall, G. Halladjian, G. D. Hallewell, K. Hamacher, P. Hamal, K. Hamano, A. Hamilton, G. N. Hamity, P. G. Hamnett, L. Han, K. Hanagaki, K. Hanawa, M. Hance, B. Haney, P. Hanke, R. Hanna, J. B. Hansen, J. D. Hansen, M. C. Hansen, P. H. Hansen, K. Hara, A. S. Hard, T. Harenberg, F. Hariri, S. Harkusha, R. D. Harrington, P. F. Harrison, F. Hartjes, M. Hasegawa, Y. Hasegawa, A. Hasib, S. Hassani, S. Haug, R. Hauser, L. Hauswald, M. Havranek, C. M. Hawkes, R. J. Hawkings, A. D. Hawkins, D. Hayden, C. P. Hays, J. M. Hays, H. S. Hayward, S. J. Haywood, S. J. Head, T. Heck, V. Hedberg, L. Heelan, S. Heim, T. Heim, B. Heinemann, J. J. Heinrich, L. Heinrich, C. Heinz, J. Hejbal, L. Helary, S. Hellman, C. Helsens, J. Henderson, R. C. W. Henderson, Y. Heng, S. Henkelmann, A. M. Henriques Correia, S. Henrot-Versille, G. H. Herbert, Y. Hernández Jiménez, G. Herten, R. Hertenberger, L. Hervas, G. G. Hesketh, N. P. Hessey, J. W. Hetherly, R. Hickling, E. Higón-Rodriguez, E. Hill, J. C. Hill, K. H. Hiller, S. J. Hillier, I. Hinchliffe, E. Hines, R. R. Hinman, M. Hirose, D. Hirschbuehl, J. Hobbs, N. Hod, M. C. Hodgkinson, P. Hodgson, A. Hoecker, M. R. Hoeferkamp, F. Hoenig, M. Hohlfeld, D. Hohn, T. R. Holmes, M. Homann, T. M. Hong, B. H. Hooberman, W. H. Hopkins, Y. Horii, A. J. Horton, J-Y. Hostachy, S. Hou, A. Hoummada, J. Howard, J. Howarth, M. Hrabovsky, I. Hristova, J. Hrivnac, T. Hryn’ova, A. Hrynevich, C. Hsu, P. J. Hsu, S.-C. Hsu, D. Hu, Q. Hu, Y. Huang, Z. Hubacek, F. Hubaut, F. Huegging, T. B. Huffman, E. W. Hughes, G. Hughes, M. Huhtinen, T. A. Hülsing, N. Huseynov, J. Huston, J. Huth, G. Iacobucci, G. Iakovidis, I. Ibragimov, L. Iconomidou-Fayard, E. Ideal, Z. Idrissi, P. Iengo, O. Igonkina, T. Iizawa, Y. Ikegami, M. Ikeno, Y. Ilchenko, D. Iliadis, N. Ilic, T. Ince, G. Introzzi, P. Ioannou, M. Iodice, K. Iordanidou, V. Ippolito, A. Irles Quiles, C. Isaksson, M. Ishino, M. Ishitsuka, R. Ishmukhametov, C. Issever, S. Istin, F. Ito, J. M. Iturbe Ponce, R. Iuppa, J. Ivarsson, W. Iwanski, H. Iwasaki, J. M. Izen, V. Izzo, S. Jabbar, B. Jackson, M. Jackson, P. Jackson, V. Jain, K. B. Jakobi, K. Jakobs, S. Jakobsen, T. Jakoubek, D. O. Jamin, D. K. Jana, E. Jansen, R. Jansky, J. Janssen, M. Janus, G. Jarlskog, N. Javadov, T. Javůrek, F. Jeanneau, L. Jeanty, J. Jejelava, G.-Y. Jeng, D. Jennens, P. Jenni, J. Jentzsch, C. Jeske, S. Jézéquel, H. Ji, J. Jia, H. Jiang, Y. Jiang, S. Jiggins, J. Jimenez Pena, S. Jin, A. Jinaru, O. Jinnouchi, P. Johansson, K. A. Johns, W. J. Johnson, K. Jon-And, G. Jones, R. W. L. Jones, S. Jones, T. J. Jones, J. Jongmanns, P. M. Jorge, J. Jovicevic, X. Ju, A. Juste Rozas, M. K. Köhler, A. Kaczmarska, M. Kado, H. Kagan, M. Kagan, S. J. Kahn, E. Kajomovitz, C. W. Kalderon, A. Kaluza, S. Kama, A. Kamenshchikov, N. Kanaya, S. Kaneti, V. A. Kantserov, J. Kanzaki, B. Kaplan, L. S. Kaplan, A. Kapliy, D. Kar, K. Karakostas, A. Karamaoun, N. Karastathis, M. J. Kareem, E. Karentzos, M. Karnevskiy, S. N. Karpov, Z. M. Karpova, K. Karthik, V. Kartvelishvili, A. N. Karyukhin, K. Kasahara, L. Kashif, R. D. Kass, A. Kastanas, Y. Kataoka, C. Kato, A. Katre, J. Katzy, K. Kawade, K. Kawagoe, T. Kawamoto, G. Kawamura, S. Kazama, V. F. Kazanin, R. Keeler, R. Kehoe, J. S. Keller, J. J. Kempster, H. Keoshkerian, O. Kepka, B. P. Kerševan, S. Kersten, R. A. Keyes, F. Khalil-zada, H. Khandanyan, A. Khanov, A. G. Kharlamov, T. J. Khoo, V. Khovanskiy, E. Khramov, J. Khubua, S. Kido, H. Y. Kim, S. H. Kim, Y. K. Kim, N. Kimura, O. M. Kind, B. T. King, M. King, S. B. King, J. Kirk, A. E. Kiryunin, T. Kishimoto, D. Kisielewska, F. Kiss, K. Kiuchi, O. Kivernyk, E. Kladiva, M. H. Klein, M. Klein, U. Klein, K. Kleinknecht, P. Klimek, A. Klimentov, R. Klingenberg, J. A. Klinger, T. Klioutchnikova, E.-E. Kluge, P. Kluit, S. Kluth, J. Knapik, E. Kneringer, E. B. F. G. Knoops, A. Knue, A. Kobayashi, D. Kobayashi, T. Kobayashi, M. Kobel, M. Kocian, P. Kodys, T. Koffas, E. Koffeman, L. A. Kogan, T. Kohriki, T. Koi, H. Kolanoski, M. Kolb, I. Koletsou, A. A. Komar, Y. Komori, T. Kondo, N. Kondrashova, K. Köneke, A. C. König, T. Kono, R. Konoplich, N. Konstantinidis, R. Kopeliansky, S. Koperny, L. Köpke, A. K. Kopp, K. Korcyl, K. Kordas, A. Korn, A. A. Korol, I. Korolkov, E. V. Korolkova, O. Kortner, S. Kortner, T. Kosek, V. V. Kostyukhin, V. M. Kotov, A. Kotwal, A. Kourkoumeli-Charalampidi, C. Kourkoumelis, V. Kouskoura, A. Koutsman, A. B. Kowalewska, R. Kowalewski, T. Z. Kowalski, W. Kozanecki, A. S. Kozhin, V. A. Kramarenko, G. Kramberger, D. Krasnopevtsev, M. W. Krasny, A. Krasznahorkay, J. K. Kraus, A. Kravchenko, M. Kretz, J. Kretzschmar, K. Kreutzfeldt, P. Krieger, K. Krizka, K. Kroeninger, H. Kroha, J. Kroll, J. Kroseberg, J. Krstic, U. Kruchonak, H. Krüger, N. Krumnack, A. Kruse, M. C. Kruse, M. Kruskal, T. Kubota, H. Kucuk, S. Kuday, J. T. Kuechler, S. Kuehn, A. Kugel, F. Kuger, A. Kuhl, T. Kuhl, V. Kukhtin, R. Kukla, Y. Kulchitsky, S. Kuleshov, M. Kuna, T. Kunigo, A. Kupco, H. Kurashige, Y. A. Kurochkin, V. Kus, E. S. Kuwertz, M. Kuze, J. Kvita, T. Kwan, D. Kyriazopoulos, A. La Rosa, J. L. La Rosa Navarro, L. La Rotonda, C. Lacasta, F. Lacava, J. Lacey, H. Lacker, D. Lacour, V. R. Lacuesta, E. Ladygin, R. Lafaye, B. Laforge, T. Lagouri, S. Lai, S. Lammers, W. Lampl, E. Lançon, U. Landgraf, M. P. J. Landon, V. S. Lang, J. C. Lange, A. J. Lankford, F. Lanni, K. Lantzsch, A. Lanza, S. Laplace, C. Lapoire, J. F. Laporte, T. Lari, F. Lasagni Manghi, M. Lassnig, P. Laurelli, W. Lavrijsen, A. T. Law, P. Laycock, T. Lazovich, M. Lazzaroni, O. Le Dortz, E. Le Guirriec, E. Le Menedeu, E. P. Le Quilleuc, M. LeBlanc, T. LeCompte, F. Ledroit-Guillon, C. A. Lee, S. C. Lee, L. Lee, G. Lefebvre, M. Lefebvre, F. Legger, C. Leggett, A. Lehan, G. Lehmann Miotto, X. Lei, W. A. Leight, A. Leisos, A. G. Leister, M. A. L. Leite, R. Leitner, D. Lellouch, B. Lemmer, K. J. C. Leney, T. Lenz, B. Lenzi, R. Leone, S. Leone, C. Leonidopoulos, S. Leontsinis, G. Lerner, C. Leroy, A. A. J. Lesage, C. G. Lester, M. Levchenko, J. Levêque, D. Levin, L. J. Levinson, M. Levy, A. M. Leyko, M. Leyton, B. Li, H. Li, H. L. Li, L. Li, L. Li, Q. Li, S. Li, X. Li, Y. Li, Z. Liang, H. Liao, B. Liberti, A. Liblong, P. Lichard, K. Lie, J. Liebal, W. Liebig, C. Limbach, A. Limosani, S. C. Lin, T. H. Lin, B. E. Lindquist, E. Lipeles, A. Lipniacka, M. Lisovyi, T. M. Liss, D. Lissauer, A. Lister, A. M. Litke, B. Liu, D. Liu, H. Liu, H. Liu, J. Liu, J. B. Liu, K. Liu, L. Liu, M. Liu, M. Liu, Y. L. Liu, Y. Liu, M. Livan, A. Lleres, J. Llorente Merino, S. L. Lloyd, F. Lo Sterzo, E. Lobodzinska, P. Loch, W. S. Lockman, F. K. Loebinger, A. E. Loevschall-Jensen, K. M. Loew, A. Loginov, T. Lohse, K. Lohwasser, M. Lokajicek, B. A. Long, J. D. Long, R. E. Long, L. Longo, K. A. Looper, L. Lopes, D. Lopez Mateos, B. Lopez Paredes, I. Lopez Paz, A. Lopez Solis, J. Lorenz, N. Lorenzo Martinez, M. Losada, P. J. Lösel, X. Lou, A. Lounis, J. Love, P. A. Love, H. Lu, N. Lu, H. J. Lubatti, C. Luci, A. Lucotte, C. Luedtke, F. Luehring, W. Lukas, L. Luminari, O. Lundberg, B. Lund-Jensen, D. Lynn, R. Lysak, E. Lytken, V. Lyubushkin, H. Ma, L. L. Ma, Y. Ma, G. Maccarrone, A. Macchiolo, C. M. Macdonald, B. Maček, J. Machado Miguens, D. Madaffari, R. Madar, H. J. Maddocks, W. F. Mader, A. Madsen, J. Maeda, S. Maeland, T. Maeno, A. Maevskiy, E. Magradze, J. Mahlstedt, C. Maiani, C. Maidantchik, A. A. Maier, T. Maier, A. Maio, S. Majewski, Y. Makida, N. Makovec, B. Malaescu, Pa. Malecki, V. P. Maleev, F. Malek, U. Mallik, D. Malon, C. Malone, S. Maltezos, V. M. Malyshev, S. Malyukov, J. Mamuzic, G. Mancini, B. Mandelli, L. Mandelli, I. Mandić, J. Maneira, L. Manhaes de Andrade Filho, J. Manjarres Ramos, A. Mann, B. Mansoulie, R. Mantifel, M. Mantoani, S. Manzoni, L. Mapelli, G. Marceca, L. March, G. Marchiori, M. Marcisovsky, M. Marjanovic, D. E. Marley, F. Marroquim, S. P. Marsden, Z. Marshall, L. F. Marti, S. Marti-Garcia, B. Martin, T. A. Martin, V. J. Martin, B. Martin dit Latour, M. Martinez, S. Martin-Haugh, V. S. Martoiu, A. C. Martyniuk, M. Marx, F. Marzano, A. Marzin, L. Masetti, T. Mashimo, R. Mashinistov, J. Masik, A. L. Maslennikov, I. Massa, L. Massa, P. Mastrandrea, A. Mastroberardino, T. Masubuchi, P. Mättig, J. Mattmann, J. Maurer, S. J. Maxfield, D. A. Maximov, R. Mazini, S. M. Mazza, N. C. Mc Fadden, G. Mc Goldrick, S. P. Mc Kee, A. McCarn, R. L. McCarthy, T. G. McCarthy, L. I. McClymont, K. W. McFarlane, J. A. Mcfayden, G. Mchedlidze, S. J. McMahon, R. A. McPherson, M. Medinnis, S. Meehan, S. Mehlhase, A. Mehta, K. Meier, C. Meineck, B. Meirose, B. R. Mellado Garcia, F. Meloni, A. Mengarelli, S. Menke, E. Meoni, K. M. Mercurio, S. Mergelmeyer, P. Mermod, L. Merola, C. Meroni, F. S. Merritt, A. Messina, J. Metcalfe, A. S. Mete, C. Meyer, C. Meyer, J-P. Meyer, J. Meyer, H. Meyer Zu Theenhausen, R. P. Middleton, S. Miglioranzi, L. Mijović, G. Mikenberg, M. Mikestikova, M. Mikuž, M. Milesi, A. Milic, D. W. Miller, C. Mills, A. Milov, D. A. Milstead, A. A. Minaenko, Y. Minami, I. A. Minashvili, A. I. Mincer, B. Mindur, M. Mineev, Y. Ming, L. M. Mir, K. P. Mistry, T. Mitani, J. Mitrevski, V. A. Mitsou, A. Miucci, P. S. Miyagawa, J. U. Mjörnmark, T. Moa, K. Mochizuki, S. Mohapatra, W. Mohr, S. Molander, R. Moles-Valls, R. Monden, M. C. Mondragon, K. Mönig, J. Monk, E. Monnier, A. Montalbano, J. Montejo Berlingen, F. Monticelli, S. Monzani, R. W. Moore, N. Morange, D. Moreno, M. Moreno Llácer, P. Morettini, D. Mori, T. Mori, M. Morii, M. Morinaga, V. Morisbak, S. Moritz, A. K. Morley, G. Mornacchi, J. D. Morris, S. S. Mortensen, L. Morvaj, M. Mosidze, J. Moss, K. Motohashi, R. Mount, E. Mountricha, S. V. Mouraviev, E. J. W. Moyse, S. Muanza, R. D. Mudd, F. Mueller, J. Mueller, R. S. P. Mueller, T. Mueller, D. Muenstermann, P. Mullen, G. A. Mullier, F. J. Munoz Sanchez, J. A. Murillo Quijada, W. J. Murray, H. Musheghyan, M. Muskinja, A. G. Myagkov, M. Myska, B. P. Nachman, O. Nackenhorst, J. Nadal, K. Nagai, R. Nagai, K. Nagano, Y. Nagasaka, K. Nagata, M. Nagel, E. Nagy, A. M. Nairz, Y. Nakahama, K. Nakamura, T. Nakamura, I. Nakano, H. Namasivayam, R. F. Naranjo Garcia, R. Narayan, D. I. Narrias Villar, I. Naryshkin, T. Naumann, G. Navarro, R. Nayyar, H. A. Neal, P. Yu. Nechaeva, T. J. Neep, P. D. Nef, A. Negri, M. Negrini, S. Nektarijevic, C. Nellist, A. Nelson, S. Nemecek, P. Nemethy, A. A. Nepomuceno, M. Nessi, M. S. Neubauer, M. Neumann, R. M. Neves, P. Nevski, P. R. Newman, D. H. Nguyen, R. B. Nickerson, R. Nicolaidou, B. Nicquevert, J. Nielsen, A. Nikiforov, V. Nikolaenko, I. Nikolic-Audit, K. Nikolopoulos, J. K. Nilsen, P. Nilsson, Y. Ninomiya, A. Nisati, R. Nisius, T. Nobe, L. Nodulman, M. Nomachi, I. Nomidis, T. Nooney, S. Norberg, M. Nordberg, N. Norjoharuddeen, O. Novgorodova, S. Nowak, M. Nozaki, L. Nozka, K. Ntekas, E. Nurse, F. Nuti, F. O’grady, D. C. O’Neil, A. A. O’Rourke, V. O’Shea, F. G. Oakham, H. Oberlack, T. Obermann, J. Ocariz, A. Ochi, I. Ochoa, J. P. Ochoa-Ricoux, S. Oda, S. Odaka, H. Ogren, A. Oh, S. H. Oh, C. C. Ohm, H. Ohman, H. Oide, H. Okawa, Y. Okumura, T. Okuyama, A. Olariu, L. F. Oleiro Seabra, S. A. Olivares Pino, D. Oliveira Damazio, A. Olszewski, J. Olszowska, A. Onofre, K. Onogi, P. U. E. Onyisi, C. J. Oram, M. J. Oreglia, Y. Oren, D. Orestano, N. Orlando, R. S. Orr, B. Osculati, R. Ospanov, G. Otero y Garzon, H. Otono, M. Ouchrif, F. Ould-Saada, A. Ouraou, K. P. Oussoren, Q. Ouyang, A. Ovcharova, M. Owen, R. E. Owen, V. E. Ozcan, N. Ozturk, K. Pachal, A. Pacheco Pages, C. Padilla Aranda, M. Pagáčová, S. Pagan Griso, F. Paige, P. Pais, K. Pajchel, G. Palacino, S. Palestini, M. Palka, D. Pallin, A. Palma, E. St. Panagiotopoulou, C. E. Pandini, J. G. Panduro Vazquez, P. Pani, S. Panitkin, D. Pantea, L. Paolozzi, Th. D. Papadopoulou, K. Papageorgiou, A. Paramonov, D. Paredes Hernandez, A. J. Parker, M. A. Parker, K. A. Parker, F. Parodi, J. A. Parsons, U. Parzefall, V. Pascuzzi, E. Pasqualucci, S. Passaggio, F. Pastore, Fr. Pastore, G. Pásztor, S. Pataraia, N. D. Patel, J. R. Pater, T. Pauly, J. Pearce, B. Pearson, L. E. Pedersen, M. Pedersen, S. Pedraza Lopez, R. Pedro, S. V. Peleganchuk, D. Pelikan, O. Penc, C. Peng, H. Peng, J. Penwell, B. S. Peralva, M. M. Perego, D. V. Perepelitsa, E. Perez Codina, L. Perini, H. Pernegger, S. Perrella, R. Peschke, V. D. Peshekhonov, K. Peters, R. F. Y. Peters, B. A. Petersen, T. C. Petersen, E. Petit, A. Petridis, C. Petridou, P. Petroff, E. Petrolo, M. Petrov, F. Petrucci, N. E. Pettersson, A. Peyaud, R. Pezoa, P. W. Phillips, G. Piacquadio, E. Pianori, A. Picazio, E. Piccaro, M. Piccinini, M. A. Pickering, R. Piegaia, J. E. Pilcher, A. D. Pilkington, A. W. J. Pin, J. Pina, M. Pinamonti, J. L. Pinfold, A. Pingel, S. Pires, H. Pirumov, M. Pitt, L. Plazak, M.-A. Pleier, V. Pleskot, E. Plotnikova, P. Plucinski, D. Pluth, R. Poettgen, L. Poggioli, D. Pohl, G. Polesello, A. Poley, A. Policicchio, R. Polifka, A. Polini, C. S. Pollard, V. Polychronakos, K. Pommès, L. Pontecorvo, B. G. Pope, G. A. Popeneciu, D. S. Popovic, A. Poppleton, S. Pospisil, K. Potamianos, I. N. Potrap, C. J. Potter, C. T. Potter, G. Poulard, J. Poveda, V. Pozdnyakov, M. E. Pozo Astigarraga, P. Pralavorio, A. Pranko, S. Prell, D. Price, L. E. Price, M. Primavera, S. Prince, M. Proissl, K. Prokofiev, F. Prokoshin, S. Protopopescu, J. Proudfoot, M. Przybycien, D. Puddu, D. Puldon, M. Purohit, P. Puzo, J. Qian, G. Qin, Y. Qin, A. Quadt, W. B. Quayle, M. Queitsch-Maitland, D. Quilty, S. Raddum, V. Radeka, V. Radescu, S. K. Radhakrishnan, P. Radloff, P. Rados, F. Ragusa, G. Rahal, J. A. Raine, S. Rajagopalan, M. Rammensee, C. Rangel-Smith, M. G. Ratti, F. Rauscher, S. Rave, T. Ravenscroft, M. Raymond, A. L. Read, N. P. Readioff, D. M. Rebuzzi, A. Redelbach, G. Redlinger, R. Reece, K. Reeves, L. Rehnisch, J. Reichert, H. Reisin, C. Rembser, H. Ren, M. Rescigno, S. Resconi, O. L. Rezanova, P. Reznicek, R. Rezvani, R. Richter, S. Richter, E. Richter-Was, O. Ricken, M. Ridel, P. Rieck, C. J. Riegel, J. Rieger, O. Rifki, M. Rijssenbeek, A. Rimoldi, L. Rinaldi, B. Ristić, E. Ritsch, I. Riu, F. Rizatdinova, E. Rizvi, C. Rizzi, S. H. Robertson, A. Robichaud-Veronneau, D. Robinson, J. E. M. Robinson, A. Robson, C. Roda, Y. Rodina, A. Rodriguez Perez, D. Rodriguez Rodriguez, S. Roe, C. S. Rogan, O. Røhne, A. Romaniouk, M. Romano, S. M. Romano Saez, E. Romero Adam, N. Rompotis, M. Ronzani, L. Roos, E. Ros, S. Rosati, K. Rosbach, P. Rose, O. Rosenthal, V. Rossetti, E. Rossi, L. P. Rossi, J. H. N. Rosten, R. Rosten, M. Rotaru, I. Roth, J. Rothberg, D. Rousseau, C. R. Royon, A. Rozanov, Y. Rozen, X. Ruan, F. Rubbo, I. Rubinskiy, V. I. Rud, M. S. Rudolph, F. Rühr, A. Ruiz-Martinez, Z. Rurikova, N. A. Rusakovich, A. Ruschke, H. L. Russell, J. P. Rutherfoord, N. Ruthmann, Y. F. Ryabov, M. Rybar, G. Rybkin, S. Ryu, A. Ryzhov, A. F. Saavedra, G. Sabato, S. Sacerdoti, H. F-W. Sadrozinski, R. Sadykov, F. Safai Tehrani, P. Saha, M. Sahinsoy, M. Saimpert, T. Saito, H. Sakamoto, Y. Sakurai, G. Salamanna, A. Salamon, J. E. Salazar Loyola, D. Salek, P. H. Sales De Bruin, D. Salihagic, A. Salnikov, J. Salt, D. Salvatore, F. Salvatore, A. Salvucci, A. Salzburger, D. Sammel, D. Sampsonidis, A. Sanchez, J. Sánchez, V. Sanchez Martinez, H. Sandaker, R. L. Sandbach, H. G. Sander, M. P. Sanders, M. Sandhoff, C. Sandoval, R. Sandstroem, D. P. C. Sankey, M. Sannino, A. Sansoni, C. Santoni, R. Santonico, H. Santos, I. Santoyo Castillo, K. Sapp, A. Sapronov, J. G. Saraiva, B. Sarrazin, O. Sasaki, Y. Sasaki, K. Sato, G. Sauvage, E. Sauvan, G. Savage, P. Savard, C. Sawyer, L. Sawyer, J. Saxon, C. Sbarra, A. Sbrizzi, T. Scanlon, D. A. Scannicchio, M. Scarcella, V. Scarfone, J. Schaarschmidt, P. Schacht, D. Schaefer, R. Schaefer, J. Schaeffer, S. Schaepe, S. Schaetzel, U. Schäfer, A. C. Schaffer, D. Schaile, R. D. Schamberger, V. Scharf, V. A. Schegelsky, D. Scheirich, M. Schernau, C. Schiavi, C. Schillo, M. Schioppa, S. Schlenker, K. Schmieden, C. Schmitt, S. Schmitt, S. Schmitz, B. Schneider, Y. J. Schnellbach, U. Schnoor, L. Schoeffel, A. Schoening, B. D. Schoenrock, E. Schopf, A. L. S. Schorlemmer, M. Schott, J. Schovancova, S. Schramm, M. Schreyer, N. Schuh, M. J. Schultens, H.-C. Schultz-Coulon, H. Schulz, M. Schumacher, B. A. Schumm, Ph. Schune, C. Schwanenberger, A. Schwartzman, T. A. Schwarz, Ph. Schwegler, H. Schweiger, Ph. Schwemling, R. Schwienhorst, J. Schwindling, T. Schwindt, G. Sciolla, F. Scuri, F. Scutti, J. Searcy, P. Seema, S. C. Seidel, A. Seiden, F. Seifert, J. M. Seixas, G. Sekhniaidze, K. Sekhon, S. J. Sekula, D. M. Seliverstov, N. Semprini-Cesari, C. Serfon, L. Serin, L. Serkin, M. Sessa, R. Seuster, H. Severini, T. Sfiligoj, F. Sforza, A. Sfyrla, E. Shabalina, N. W. Shaikh, L. Y. Shan, R. Shang, J. T. Shank, M. Shapiro, P. B. Shatalov, K. Shaw, S. M. Shaw, A. Shcherbakova, C. Y. Shehu, P. Sherwood, L. Shi, S. Shimizu, C. O. Shimmin, M. Shimojima, M. Shiyakova, A. Shmeleva, D. Shoaleh Saadi, M. J. Shochet, S. Shojaii, S. Shrestha, E. Shulga, M. A. Shupe, P. Sicho, P. E. Sidebo, O. Sidiropoulou, D. Sidorov, A. Sidoti, F. Siegert, Dj. Sijacki, J. Silva, S. B. Silverstein, V. Simak, O. Simard, Lj. Simic, S. Simion, E. Simioni, B. Simmons, D. Simon, M. Simon, P. Sinervo, N. B. Sinev, M. Sioli, G. Siragusa, S. Yu. Sivoklokov, J. Sjölin, T. B. Sjursen, M. B. Skinner, H. P. Skottowe, P. Skubic, M. Slater, T. Slavicek, M. Slawinska, K. Sliwa, R. Slovak, V. Smakhtin, B. H. Smart, L. Smestad, S. Yu. Smirnov, Y. Smirnov, L. N. Smirnova, O. Smirnova, M. N. K. Smith, R. W. Smith, M. Smizanska, K. Smolek, A. A. Snesarev, G. Snidero, S. Snyder, R. Sobie, F. Socher, A. Soffer, D. A. Soh, G. Sokhrannyi, C. A. Solans Sanchez, M. Solar, E. Yu. Soldatov, U. Soldevila, A. A. Solodkov, A. Soloshenko, O. V. Solovyanov, V. Solovyev, P. Sommer, H. Son, H. Y. Song, A. Sood, A. Sopczak, V. Sopko, V. Sorin, D. Sosa, C. L. Sotiropoulou, R. Soualah, A. M. Soukharev, D. South, B. C. Sowden, S. Spagnolo, M. Spalla, M. Spangenberg, F. Spanò, D. Sperlich, F. Spettel, R. Spighi, G. Spigo, L. A. Spiller, M. Spousta, R. D. St. Denis, A. Stabile, S. Staerz, J. Stahlman, R. Stamen, S. Stamm, E. Stanecka, R. W. Stanek, C. Stanescu, M. Stanescu-Bellu, M. M. Stanitzki, S. Stapnes, E. A. Starchenko, G. H. Stark, J. Stark, P. Staroba, P. Starovoitov, R. Staszewski, P. Steinberg, B. Stelzer, H. J. Stelzer, O. Stelzer-Chilton, H. Stenzel, G. A. Stewart, J. A. Stillings, M. C. Stockton, M. Stoebe, G. Stoicea, P. Stolte, S. Stonjek, A. R. Stradling, A. Straessner, M. E. Stramaglia, J. Strandberg, S. Strandberg, A. Strandlie, M. Strauss, P. Strizenec, R. Ströhmer, D. M. Strom, R. Stroynowski, A. Strubig, S. A. Stucci, B. Stugu, N. A. Styles, D. Su, J. Su, R. Subramaniam, S. Suchek, Y. Sugaya, M. Suk, V. V. Sulin, S. Sultansoy, T. Sumida, S. Sun, X. Sun, J. E. Sundermann, K. Suruliz, G. Susinno, M. R. Sutton, S. Suzuki, M. Svatos, M. Swiatlowski, I. Sykora, T. Sykora, D. Ta, C. Taccini, K. Tackmann, J. Taenzer, A. Taffard, R. Tafirout, N. Taiblum, H. Takai, R. Takashima, H. Takeda, T. Takeshita, Y. Takubo, M. Talby, A. A. Talyshev, J. Y. C. Tam, K. G. Tan, J. Tanaka, R. Tanaka, S. Tanaka, B. B. Tannenwald, S. Tapia Araya, S. Tapprogge, S. Tarem, G. F. Tartarelli, P. Tas, M. Tasevsky, T. Tashiro, E. Tassi, A. Tavares Delgado, Y. Tayalati, A. C. Taylor, G. N. Taylor, P. T. E. Taylor, W. Taylor, F. A. Teischinger, P. Teixeira-Dias, K. K. Temming, D. Temple, H. Ten Kate, P. K. Teng, J. J. Teoh, F. Tepel, S. Terada, K. Terashi, J. Terron, S. Terzo, M. Testa, R. J. Teuscher, T. Theveneaux-Pelzer, J. P. Thomas, J. Thomas-Wilsker, E. N. Thompson, P. D. Thompson, R. J. Thompson, A. S. Thompson, L. A. Thomsen, E. Thomson, M. Thomson, M. J. Tibbetts, R. E. Ticse Torres, V. O. Tikhomirov, Yu. A. Tikhonov, S. Timoshenko, P. Tipton, S. Tisserant, K. Todome, T. Todorov, S. Todorova-Nova, J. Tojo, S. Tokár, K. Tokushuku, E. Tolley, L. Tomlinson, M. Tomoto, L. Tompkins, K. Toms, B. Tong, E. Torrence, H. Torres, E. Torró Pastor, J. Toth, F. Touchard, D. R. Tovey, T. Trefzger, L. Tremblet, A. Tricoli, I. M. Trigger, S. Trincaz-Duvoid, M. F. Tripiana, W. Trischuk, B. Trocmé, A. Trofymov, C. Troncon, M. Trottier-McDonald, M. Trovatelli, L. Truong, M. Trzebinski, A. Trzupek, J. C-L. Tseng, P. V. Tsiareshka, G. Tsipolitis, N. Tsirintanis, S. Tsiskaridze, V. Tsiskaridze, E. G. Tskhadadze, K. M. Tsui, I. I. Tsukerman, V. Tsulaia, S. Tsuno, D. Tsybychev, A. Tudorache, V. Tudorache, A. N. Tuna, S. A. Tupputi, S. Turchikhin, D. Turecek, D. Turgeman, R. Turra, A. J. Turvey, P. M. Tuts, M. Tyndel, G. Ucchielli, I. Ueda, R. Ueno, M. Ughetto, F. Ukegawa, G. Unal, A. Undrus, G. Unel, F. C. Ungaro, Y. Unno, C. Unverdorben, J. Urban, P. Urquijo, P. Urrejola, G. Usai, A. Usanova, L. Vacavant, V. Vacek, B. Vachon, C. Valderanis, E. Valdes Santurio, N. Valencic, S. Valentinetti, A. Valero, L. Valery, S. Valkar, S. Vallecorsa, J. A. Valls Ferrer, W. Van Den Wollenberg, P. C. Van Der Deijl, R. van der Geer, H. van der Graaf, N. van Eldik, P. van Gemmeren, J. Van Nieuwkoop, I. van Vulpen, M. C. van Woerden, M. Vanadia, W. Vandelli, R. Vanguri, A. Vaniachine, P. Vankov, G. Vardanyan, R. Vari, E. W. Varnes, T. Varol, D. Varouchas, A. Vartapetian, K. E. Varvell, J. G. Vasquez, F. Vazeille, T. Vazquez Schroeder, J. Veatch, L. M. Veloce, F. Veloso, S. Veneziano, A. Ventura, M. Venturi, N. Venturi, A. Venturini, V. Vercesi, M. Verducci, W. Verkerke, J. C. Vermeulen, A. Vest, M. C. Vetterli, O. Viazlo, I. Vichou, T. Vickey, O. E. Vickey Boeriu, G. H. A. Viehhauser, S. Viel, L. Vigani, R. Vigne, M. Villa, M. Villaplana Perez, E. Vilucchi, M. G. Vincter, V. B. Vinogradov, C. Vittori, I. Vivarelli, S. Vlachos, M. Vlasak, M. Vogel, P. Vokac, G. Volpi, M. Volpi, H. von der Schmitt, E. von Toerne, V. Vorobel, K. Vorobev, M. Vos, R. Voss, J. H. Vossebeld, N. Vranjes, M. Vranjes Milosavljevic, V. Vrba, M. Vreeswijk, R. Vuillermet, I. Vukotic, Z. Vykydal, P. Wagner, W. Wagner, H. Wahlberg, S. Wahrmund, J. Wakabayashi, J. Walder, R. Walker, W. Walkowiak, V. Wallangen, C. Wang, C. Wang, F. Wang, H. Wang, H. Wang, J. Wang, J. Wang, K. Wang, R. Wang, S. M. Wang, T. Wang, T. Wang, X. Wang, C. Wanotayaroj, A. Warburton, C. P. Ward, D. R. Wardrope, A. Washbrook, P. M. Watkins, A. T. Watson, I. J. Watson, M. F. Watson, G. Watts, S. Watts, B. M. Waugh, S. Webb, M. S. Weber, S. W. Weber, J. S. Webster, A. R. Weidberg, B. Weinert, J. Weingarten, C. Weiser, H. Weits, P. S. Wells, T. Wenaus, T. Wengler, S. Wenig, N. Wermes, M. Werner, P. Werner, M. Wessels, J. Wetter, K. Whalen, N. L. Whallon, A. M. Wharton, A. White, M. J. White, R. White, S. White, D. Whiteson, F. J. Wickens, W. Wiedenmann, M. Wielers, P. Wienemann, C. Wiglesworth, L. A. M. Wiik-Fuchs, A. Wildauer, F. Wilk, H. G. Wilkens, H. H. Williams, S. Williams, C. Willis, S. Willocq, J. A. Wilson, I. Wingerter-Seez, F. Winklmeier, O. J. Winston, B. T. Winter, M. Wittgen, J. Wittkowski, S. J. Wollstadt, M. W. Wolter, H. Wolters, B. K. Wosiek, J. Wotschack, M. J. Woudstra, K. W. Wozniak, M. Wu, M. Wu, S. L. Wu, X. Wu, Y. Wu, T. R. Wyatt, B. M. Wynne, S. Xella, D. Xu, L. Xu, B. Yabsley, S. Yacoob, R. Yakabe, D. Yamaguchi, Y. Yamaguchi, A. Yamamoto, S. Yamamoto, T. Yamanaka, K. Yamauchi, Y. Yamazaki, Z. Yan, H. Yang, H. Yang, Y. Yang, Z. Yang, W-M. Yao, Y. C. Yap, Y. Yasu, E. Yatsenko, K. H. Yau Wong, J. Ye, S. Ye, I. Yeletskikh, A. L. Yen, E. Yildirim, K. Yorita, R. Yoshida, K. Yoshihara, C. Young, C. J. S. Young, S. Youssef, D. R. Yu, J. Yu, J. M. Yu, J. Yu, L. Yuan, S. P. Y. Yuen, I. Yusuff, B. Zabinski, R. Zaidan, A. M. Zaitsev, N. Zakharchuk, J. Zalieckas, A. Zaman, S. Zambito, L. Zanello, D. Zanzi, C. Zeitnitz, M. Zeman, A. Zemla, J. C. Zeng, Q. Zeng, K. Zengel, O. Zenin, T. Ženiš, D. Zerwas, D. Zhang, F. Zhang, G. Zhang, H. Zhang, J. Zhang, L. Zhang, R. Zhang, R. Zhang, X. Zhang, Z. Zhang, X. Zhao, Y. Zhao, Z. Zhao, A. Zhemchugov, J. Zhong, B. Zhou, C. Zhou, L. Zhou, L. Zhou, M. Zhou, N. Zhou, C. G. Zhu, H. Zhu, J. Zhu, Y. Zhu, X. Zhuang, K. Zhukov, A. Zibell, D. Zieminska, N. I. Zimine, C. Zimmermann, S. Zimmermann, Z. Zinonos, M. Zinser, M. Ziolkowski, L. Živković, G. Zobernig, A. Zoccoli, M. zur Nedden, G. Zurzolo, L. Zwalinski

**Affiliations:** 1 Department of Physics, University of Adelaide, Adelaide, Australia; 2 Physics Department, SUNY Albany, Albany, NY USA; 3 Department of Physics, University of Alberta, Edmonton, AB Canada; 4 Department of Physics, Ankara University, Ankara, Turkey; 5 Istanbul Aydin University, Istanbul, Turkey; 6 Division of Physics, TOBB University of Economics and Technology, Ankara, Turkey; 7 LAPP, CNRS/IN2P3 and Université Savoie Mont Blanc, Annecy-le-Vieux, France; 8 High Energy Physics Division, Argonne National Laboratory, Argonne, IL USA; 9 Department of Physics, University of Arizona, Tucson, AZ USA; 10 Department of Physics, The University of Texas at Arlington, Arlington, TX USA; 11 Physics Department, University of Athens, Athens, Greece; 12 Physics Department, National Technical University of Athens, Zografou, Greece; 13 Institute of Physics, Azerbaijan Academy of Sciences, Baku, Azerbaijan; 14 Institut de Física d’Altes Energies (IFAE), The Barcelona Institute of Science and Technology, Barcelona, Spain; 15 Institute of Physics, University of Belgrade, Belgrade, Serbia; 16 Department for Physics and Technology, University of Bergen, Bergen, Norway; 17 Physics Division, Lawrence Berkeley National Laboratory and University of California, Berkeley, CA USA; 18 Department of Physics, Humboldt University, Berlin, Germany; 19 Albert Einstein Center for Fundamental Physics and Laboratory for High Energy Physics, University of Bern, Bern, Switzerland; 20 School of Physics and Astronomy, University of Birmingham, Birmingham, UK; 21 Department of Physics, Bogazici University, Istanbul, Turkey; 22 Department of Physics Engineering, Gaziantep University, Gaziantep, Turkey; 23 Faculty of Engineering and Natural Sciences, Istanbul Bilgi University, Istanbul, Turkey; 24 Faculty of Engineering and Natural Sciences, Bahcesehir University, Istanbul, Turkey; 25 Centro de Investigaciones, Universidad Antonio Narino, Bogotá, Colombia; 26 INFN Sezione di Bologna, Bologna, Italy; 27 Dipartimento di Fisica e Astronomia, Università di Bologna, Bologna, Italy; 28 Physikalisches Institut, University of Bonn, Bonn, Germany; 29 Department of Physics, Boston University, Boston, MA USA; 30 Department of Physics, Brandeis University, Waltham, MA USA; 31 Universidade Federal do Rio De Janeiro COPPE/EE/IF, Rio de Janeiro, Brazil; 32 Electrical Circuits Department, Federal University of Juiz de Fora (UFJF), Juiz de Fora, Brazil; 33 Federal University of Sao Joao del Rei (UFSJ), Sao Joao del Rei, Brazil; 34 Instituto de Fisica, Universidade de Sao Paulo, São Paulo, Brazil; 35 Physics Department, Brookhaven National Laboratory, Upton, NY USA; 36 Transilvania University of Brasov, Brasov, Romania; 37 National Institute of Physics and Nuclear Engineering, Bucharest, Romania; 38 Physics Department, National Institute for Research and Development of Isotopic and Molecular Technologies, Cluj Napoca, Romania; 39 University Politehnica Bucharest, Bucharest, Romania; 40 West University in Timisoara, Timisoara, Romania; 41 Departamento de Física, Universidad de Buenos Aires, Buenos Aires, Argentina; 42 Cavendish Laboratory, University of Cambridge, Cambridge, UK; 43 Department of Physics, Carleton University, Ottawa, ON Canada; 44 CERN, Geneva, Switzerland; 45 Enrico Fermi Institute, University of Chicago, Chicago, IL USA; 46 Departamento de Física, Pontificia Universidad Católica de Chile, Santiago, Chile; 47 Departamento de Física, Universidad Técnica Federico Santa María, Valparaiso, Chile; 48 Institute of High Energy Physics, Chinese Academy of Sciences, Beijing, China; 49 Department of Modern Physics, University of Science and Technology of China, Hefei, Anhui China; 50 Department of Physics, Nanjing University, Nanjing, Jiangsu China; 51 School of Physics, Shandong University, Jinan, Shandong China; 52 Shanghai Key Laboratory for Particle Physics and Cosmology, Department of Physics and Astronomy, Shanghai Jiao Tong University, (also affiliated with PKU-CHEP), Shanghai, China; 53 Physics Department, Tsinghua University, Beijing, 100084 China; 54 Laboratoire de Physique Corpusculaire, Clermont Université and Université Blaise Pascal and CNRS/IN2P3, Clermont-Ferrand, France; 55 Nevis Laboratory, Columbia University, Irvington, NY USA; 56 Niels Bohr Institute, University of Copenhagen, Copenhagen, Denmark; 57 INFN Gruppo Collegato di Cosenza, Laboratori Nazionali di Frascati, Frascati, Italy; 58 Dipartimento di Fisica, Università della Calabria, Rende, Italy; 59 Faculty of Physics and Applied Computer Science, AGH University of Science and Technology, Kraków, Poland; 60 Marian Smoluchowski Institute of Physics, Jagiellonian University, Kraków, Poland; 61 Institute of Nuclear Physics, Polish Academy of Sciences, Kraków, Poland; 62 Physics Department, Southern Methodist University, Dallas, TX USA; 63 Physics Department, University of Texas at Dallas, Richardson, TX USA; 64 DESY, Hamburg and Zeuthen, Germany; 65 Institut für Experimentelle Physik IV, Technische Universität Dortmund, Dortmund, Germany; 66 Institut für Kern- und Teilchenphysik, Technische Universität Dresden, Dresden, Germany; 67 Department of Physics, Duke University, Durham, NC USA; 68 SUPA-School of Physics and Astronomy, University of Edinburgh, Edinburgh, UK; 69 INFN Laboratori Nazionali di Frascati, Frascati, Italy; 70 Fakultät für Mathematik und Physik, Albert-Ludwigs-Universität, Freiburg, Germany; 71 Section de Physique, Université de Genève, Geneva, Switzerland; 72 INFN Sezione di Genova, Genoa, Italy; 73 Dipartimento di Fisica, Università di Genova, Genoa, Italy; 74 E. Andronikashvili Institute of Physics, Iv. Javakhishvili Tbilisi State University, Tbilisi, Georgia; 75 High Energy Physics Institute, Tbilisi State University, Tbilisi, Georgia; 76 II Physikalisches Institut, Justus-Liebig-Universität Giessen, Giessen, Germany; 77 SUPA-School of Physics and Astronomy, University of Glasgow, Glasgow, UK; 78 II Physikalisches Institut, Georg-August-Universität, Göttingen, Germany; 79 Laboratoire de Physique Subatomique et de Cosmologie, Université Grenoble-Alpes, CNRS/IN2P3, Grenoble, France; 80 Department of Physics, Hampton University, Hampton, VA USA; 81 Laboratory for Particle Physics and Cosmology, Harvard University, Cambridge, MA USA; 82 Kirchhoff-Institut für Physik, Ruprecht-Karls-Universität Heidelberg, Heidelberg, Germany; 83 Physikalisches Institut, Ruprecht-Karls-Universität Heidelberg, Heidelberg, Germany; 84 ZITI Institut für technische Informatik, Ruprecht-Karls-Universität Heidelberg, Mannheim, Germany; 85 Faculty of Applied Information Science, Hiroshima Institute of Technology, Hiroshima, Japan; 86 Department of Physics, The Chinese University of Hong Kong, Shatin, NT Hong Kong; 87 Department of Physics, The University of Hong Kong, Hong Kong, China; 88 Department of Physics, The Hong Kong University of Science and Technology, Clear Water Bay, Kowloon, Hong Kong, China; 89 Department of Physics, Indiana University, Bloomington, IN USA; 90 Institut für Astro- und Teilchenphysik, Leopold-Franzens-Universität, Innsbruck, Austria; 91 University of Iowa, Iowa City, IA USA; 92 Department of Physics and Astronomy, Iowa State University, Ames, IA USA; 93 Joint Institute for Nuclear Research, JINR Dubna, Dubna, Russia; 94 KEK, High Energy Accelerator Research Organization, Tsukuba, Japan; 95 Graduate School of Science, Kobe University, Kobe, Japan; 96 Faculty of Science, Kyoto University, Kyoto, Japan; 97 Kyoto University of Education, Kyoto, Japan; 98 Department of Physics, Kyushu University, Fukuoka, Japan; 99 Instituto de Física La Plata, Universidad Nacional de La Plata and CONICET, La Plata, Argentina; 100 Physics Department, Lancaster University, Lancaster, UK; 101 INFN Sezione di Lecce, Lecce, Italy; 102 Dipartimento di Matematica e Fisica, Università del Salento, Lecce, Italy; 103 Oliver Lodge Laboratory, University of Liverpool, Liverpool, UK; 104 Department of Physics, Jožef Stefan Institute and University of Ljubljana, Ljubljana, Slovenia; 105 School of Physics and Astronomy, Queen Mary University of London, London, UK; 106 Department of Physics, Royal Holloway University of London, Surrey, UK; 107 Department of Physics and Astronomy, University College London, London, UK; 108 Louisiana Tech University, Ruston, LA USA; 109 Laboratoire de Physique Nucléaire et de Hautes Energies, UPMC and Université Paris-Diderot and CNRS/IN2P3, Paris, France; 110 Fysiska institutionen, Lunds universitet, Lund, Sweden; 111 Departamento de Fisica Teorica C-15, Universidad Autonoma de Madrid, Madrid, Spain; 112 Institut für Physik, Universität Mainz, Mainz, Germany; 113 School of Physics and Astronomy, University of Manchester, Manchester, UK; 114 CPPM, Aix-Marseille Université and CNRS/IN2P3, Marseille, France; 115 Department of Physics, University of Massachusetts, Amherst, MA USA; 116 Department of Physics, McGill University, Montreal, QC Canada; 117 School of Physics, University of Melbourne, Melbourne, VIC Australia; 118 Department of Physics, The University of Michigan, Ann Arbor, MI USA; 119 Department of Physics and Astronomy, Michigan State University, East Lansing, MI USA; 120 INFN Sezione di Milano, Milan, Italy; 121 Dipartimento di Fisica, Università di Milano, Milan, Italy; 122 B.I. Stepanov Institute of Physics, National Academy of Sciences of Belarus, Minsk, Republic of Belarus; 123 National Scientific and Educational Centre for Particle and High Energy Physics, Minsk, Republic of Belarus; 124 Group of Particle Physics, University of Montreal, Montreal, QC Canada; 125 P.N. Lebedev Physical Institute of the Russian, Academy of Sciences, Moscow, Russia; 126 Institute for Theoretical and Experimental Physics (ITEP), Moscow, Russia; 127 National Research Nuclear University MEPhI, Moscow, Russia; 128 D.V. Skobeltsyn Institute of Nuclear Physics, M.V. Lomonosov Moscow State University, Moscow, Russia; 129 Fakultät für Physik, Ludwig-Maximilians-Universität München, Munich, Germany; 130 Max-Planck-Institut für Physik (Werner-Heisenberg-Institut), Munich, Germany; 131 Nagasaki Institute of Applied Science, Nagasaki, Japan; 132 Graduate School of Science and Kobayashi-Maskawa Institute, Nagoya University, Nagoya, Japan; 133 INFN Sezione di Napoli, Naples, Italy; 134 Dipartimento di Fisica, Università di Napoli, Naples, Italy; 135 Department of Physics and Astronomy, University of New Mexico, Albuquerque, NM USA; 136 Institute for Mathematics, Astrophysics and Particle Physics, Radboud University Nijmegen/Nikhef, Nijmegen, The Netherlands; 137 Nikhef National Institute for Subatomic Physics and University of Amsterdam, Amsterdam, The Netherlands; 138 Department of Physics, Northern Illinois University, DeKalb, IL USA; 139 Budker Institute of Nuclear Physics, SB RAS, Novosibirsk, Russia; 140 Department of Physics, New York University, New York, NY USA; 141 Ohio State University, Columbus, OH USA; 142 Faculty of Science, Okayama University, Okayama, Japan; 143 Homer L. Dodge Department of Physics and Astronomy, University of Oklahoma, Norman, OK USA; 144 Department of Physics, Oklahoma State University, Stillwater, OK USA; 145 Palacký University, RCPTM, Olomouc, Czech Republic; 146 Center for High Energy Physics, University of Oregon, Eugene, OR USA; 147 LAL, Univ. Paris-Sud, CNRS/IN2P3, Université Paris Saclay, Orsay, France; 148 Graduate School of Science, Osaka University, Osaka, Japan; 149 Department of Physics, University of Oslo, Oslo, Norway; 150 Department of Physics, Oxford University, Oxford, UK; 151 INFN Sezione di Pavia, Pavia, Italy; 152 Dipartimento di Fisica, Università di Pavia, Pavia, Italy; 153 Department of Physics, University of Pennsylvania, Philadelphia, PA USA; 154 National Research Centre “Kurchatov Institute” B.P.Konstantinov Petersburg Nuclear Physics Institute, St. Petersburg, Russia; 155 INFN Sezione di Pisa, Pisa, Italy; 156 Dipartimento di Fisica E. Fermi, Università di Pisa, Pisa, Italy; 157 Department of Physics and Astronomy, University of Pittsburgh, Pittsburgh, PA USA; 158 Laboratório de Instrumentação e Física Experimental de Partículas-LIP, Lisbon, Portugal; 159 Faculdade de Ciências, Universidade de Lisboa, Lisbon, Portugal; 160 Department of Physics, University of Coimbra, Coimbra, Portugal; 161 Centro de Física Nuclear da Universidade de Lisboa, Lisbon, Portugal; 162 Departamento de Fisica, Universidade do Minho, Braga, Portugal; 163 Departamento de Fisica Teorica y del Cosmos and CAFPE, Universidad de Granada, Granada, Spain; 164 Dep Fisica and CEFITEC of Faculdade de Ciencias e Tecnologia, Universidade Nova de Lisboa, Caparica, Portugal; 165 Institute of Physics, Academy of Sciences of the Czech Republic, Prague, Czech Republic; 166 Czech Technical University in Prague, Prague, Czech Republic; 167 Faculty of Mathematics and Physics, Charles University in Prague, Prague, Czech Republic; 168 State Research Center Institute for High Energy Physics (Protvino), NRC KI, Protvino, Russia; 169 Particle Physics Department, Rutherford Appleton Laboratory, Didcot, UK; 170 INFN Sezione di Roma, Rome, Italy; 171 Dipartimento di Fisica, Sapienza Università di Roma, Rome, Italy; 172 INFN Sezione di Roma Tor Vergata, Rome, Italy; 173 Dipartimento di Fisica, Università di Roma Tor Vergata, Rome, Italy; 174 INFN Sezione di Roma Tre, Rome, Italy; 175 Dipartimento di Matematica e Fisica, Università Roma Tre, Rome, Italy; 176 Faculté des Sciences Ain Chock, Réseau Universitaire de Physique des Hautes Energies-Université Hassan II, Casablanca, Morocco; 177 Centre National de l’Energie des Sciences Techniques Nucleaires, Rabat, Morocco; 178 Faculté des Sciences Semlalia, Université Cadi Ayyad, LPHEA-Marrakech, Marrakech, Morocco; 179 Faculté des Sciences, Université Mohamed Premier and LPTPM, Oujda, Morocco; 180 Faculté des Sciences, Université Mohammed V, Rabat, Morocco; 181 DSM/IRFU (Institut de Recherches sur les Lois Fondamentales de l’Univers), CEA Saclay (Commissariat à l’Energie Atomique et aux Energies Alternatives), Gif-sur-Yvette, France; 182 Santa Cruz Institute for Particle Physics, University of California Santa Cruz, Santa Cruz, CA USA; 183 Department of Physics, University of Washington, Seattle, WA USA; 184 Department of Physics and Astronomy, University of Sheffield, Sheffield, UK; 185 Department of Physics, Shinshu University, Nagano, Japan; 186 Fachbereich Physik, Universität Siegen, Siegen, Germany; 187 Department of Physics, Simon Fraser University, Burnaby, BC Canada; 188 SLAC National Accelerator Laboratory, Stanford, CA USA; 189 Faculty of Mathematics, Physics and Informatics, Comenius University, Bratislava, Slovak Republic; 190 Department of Subnuclear Physics, Institute of Experimental Physics of the Slovak Academy of Sciences, Kosice, Slovak Republic; 191 Department of Physics, University of Cape Town, Cape Town, South Africa; 192 Department of Physics, University of Johannesburg, Johannesburg, South Africa; 193 School of Physics, University of the Witwatersrand, Johannesburg, South Africa; 194 Department of Physics, Stockholm University, Stockholm, Sweden; 195 The Oskar Klein Centre, Stockholm, Sweden; 196 Physics Department, Royal Institute of Technology, Stockholm, Sweden; 197 Departments of Physics and Astronomy and Chemistry, Stony Brook University, Stony Brook, NY USA; 198 Department of Physics and Astronomy, University of Sussex, Brighton, UK; 199 School of Physics, University of Sydney, Sydney, Australia; 200 Institute of Physics, Academia Sinica, Taipei, Taiwan; 201 Department of Physics, Technion: Israel Institute of Technology, Haifa, Israel; 202 Raymond and Beverly Sackler School of Physics and Astronomy, Tel Aviv University, Tel Aviv, Israel; 203 Department of Physics, Aristotle University of Thessaloniki, Thessaloníki, Greece; 204 International Center for Elementary Particle Physics and Department of Physics, The University of Tokyo, Tokyo, Japan; 205 Graduate School of Science and Technology, Tokyo Metropolitan University, Tokyo, Japan; 206 Department of Physics, Tokyo Institute of Technology, Tokyo, Japan; 207 Department of Physics, University of Toronto, Toronto, ON Canada; 208 TRIUMF, Vancouver, BC Canada; 209 Department of Physics and Astronomy, York University, Toronto, ON Canada; 210 Faculty of Pure and Applied Sciences, and Center for Integrated Research in Fundamental Science and Engineering, University of Tsukuba, Tsukuba, Japan; 211 Department of Physics and Astronomy, Tufts University, Medford, MA USA; 212 Department of Physics and Astronomy, University of California Irvine, Irvine, CA USA; 213 INFN Gruppo Collegato di Udine, Sezione di Trieste, Udine, Italy; 214 ICTP, Trieste, Italy; 215 Dipartimento di Chimica Fisica e Ambiente, Università di Udine, Udine, Italy; 216 Department of Physics and Astronomy, University of Uppsala, Uppsala, Sweden; 217 Department of Physics, University of Illinois, Urbana, IL USA; 218 Instituto de Física Corpuscular (IFIC) and Departamento de Física Atómica, Molecular y Nuclear and Departamento de Ingeniería Electrónica and Instituto de Microelectrónica de Barcelona (IMB-CNM), University of Valencia and CSIC, Valencia, Spain; 219 Department of Physics, University of British Columbia, Vancouver, BC Canada; 220 Department of Physics and Astronomy, University of Victoria, Victoria, BC Canada; 221 Department of Physics, University of Warwick, Coventry, UK; 222 Waseda University, Tokyo, Japan; 223 Department of Particle Physics, The Weizmann Institute of Science, Rehovot, Israel; 224 Department of Physics, University of Wisconsin, Madison, WI USA; 225 Fakultät für Physik und Astronomie, Julius-Maximilians-Universität, Würzburg, Germany; 226 Fakultät für Mathematik und Naturwissenschaften, Fachgruppe Physik, Bergische Universität Wuppertal, Wuppertal, Germany; 227 Department of Physics, Yale University, New Haven, CT USA; 228 Yerevan Physics Institute, Yerevan, Armenia; 229 Centre de Calcul de l’Institut National de Physique Nucléaire et de Physique des Particules (IN2P3), Villeurbanne, France; 230 CERN, Geneva, Switzerland

## Abstract

Event-shape observables measured using charged particles in inclusive *Z*-boson events are presented, using the electron and muon decay modes of the *Z* bosons. The measurements are based on an integrated luminosity of $1.1~\mathrm{fb}^{-1}$ of proton–proton collisions recorded by the ATLAS detector at the LHC at a centre-of-mass energy $\sqrt{s}=7$ ${\mathrm{TeV}}$. Charged-particle distributions, excluding the lepton–antilepton pair from the *Z*-boson decay, are measured in different ranges of transverse momentum of the *Z* boson. Distributions include multiplicity, scalar sum of transverse momenta, beam thrust, transverse thrust, spherocity, and ${\mathcal {F}}$-parameter, which are in particular sensitive to properties of the underlying event at small values of the *Z*-boson transverse momentum. The measured observables are compared with predictions from Pythia 8, Sherpa, and Herwig 7. Typically, all three Monte Carlo generators provide predictions that are in better agreement with the data at high *Z*-boson transverse momenta than at low *Z*-boson transverse momenta, and for the observables that are less sensitive to the number of charged particles in the event.

## Introduction

The Large Hadron Collider (LHC) was primarily built to explore the mechanism of electroweak symmetry breaking and to search for new physics beyond the Standard Model (SM) in proton–proton collisions characterised by parton–parton scatterings with a high momentum transfer. These parton–parton scatterings are unavoidably accompanied by interactions between the proton remnants which are often called the “underlying event” (UE) and have to be modelled well in order to be able to measure high-momentum-transfer processes to high accuracy.

Since the UE is dominated by low-scale strong-force interactions, in which the strong coupling strength diverges and perturbative methods of quantum chromodynamics (QCD) lose predictivity, it is extremely difficult to predict UE-sensitive observables from an ab-initio calculation in QCD. As a result, one has to rely on models implemented in general-purpose Monte Carlo (MC) event generators. Generators such as Herwig 7  [1], Pythia 8  [2], and Sherpa  [3] contain multiple partonic interactions (MPI) as well as QCD radiation in the initial and final state to describe the UE. Certain aspects of the UE, e.g. the average transverse momenta of charged particles as a function of the charged-particle multiplicity, are better modelled by introducing in addition a mechanism of colour reconnection as in the event generators Pythia 8 and Herwig++  [4, 5]/Herwig 7. Such a mechanism is also implemented in Sherpa, but not activated by default and not used in ATLAS simulations using Sherpa. It is impossible to unambiguously separate the UE from the hard scattering process on an event-by-event basis. However, distributions can be measured that are particularly sensitive to the properties of the UE. Such measurements have been performed in proton–antiproton collisions in jet and in Drell–Yan production by the CDF experiment [6, 7] at centre-of-mass energies $\sqrt{s} =$ 1.8 and 1.96 TeV, and in proton–proton collisions at $\sqrt{s}=900$ GeV and 7 TeV by the ATLAS experiment [8–13], the ALICE experiment [14] and the CMS experiment [15–17].

This paper presents an analysis of event-shape observables sensitive to UE properties in 7 ${\mathrm{TeV}}$ proton–proton collisions at the LHC. The dataset of $1.1~{\text {fb}}^{-1}$ integrated luminosity was collected by the ATLAS detector [18] during data-taking in 2011, and events were selected by requiring a *Z*-boson candidate decaying to an $e^{+}e^{-}$ or $\mu ^{+}\mu ^{-}$ pair. Since the *Z* boson is an object without colour charge, it does not affect hadronic activity in the collision and the observables were calculated using charged particles excluding the *Z*-boson decay products. The charged-particle event-shape observables beam thrust, transverse thrust, spherocity, and ${\mathcal {F}}$-parameter as defined in Sect. 2 were measured in inclusive *Z* production. This paper contains information about aspects of the UE which were not explored by previous studies. The transverse thrust event-shape variable was measured by the CMS experiment [19] in *Z* events with at least one hard jet, with the goal of testing predictions from perturbative QCD. Since different hard process scales have different sensitivities to different aspects of the UE modelling, the observables were measured in the present paper in different ranges of the transverse momentum^1^ of the *Z*-boson candidate, $p_\text {T}(\ell ^{+}\ell ^{-})$.^2^ At small $p_\text {T}(\ell ^{+}\ell ^{-})$ values, events are expected to have low jet activity from the hard process and hence high sensitivity to UE characteristics. At high $p_\text {T}(\ell ^{+}\ell ^{-})$ values, the event is expected to contain at least one jet of high transverse momentum recoiling against the $\ell ^{+}\ell ^{-}$ system, which is expected to be reasonably described by perturbative calculations of the hard process.

The measured distributions have been corrected for the effects of pile-up (PU), which are additional proton–proton interactions in the same LHC bunch crossing, for detector effects, and for the dominant background contribution from multijet events. The results are compared with the predictions of the MC event generators Pythia 8, Herwig 7, and Sherpa.

The paper is organised as follows: Sect. 2 introduces the event-shape observables and defines the particle-level phase space used in this measurement. Sections 3 and 4 describe the ATLAS detector and the Monte Carlo event generators relevant to this analysis, which is described in detail in Sect. 5. The results are presented and discussed in Sect. 6 and summarised in Sect. 7.

## Event-shape observables

The observables were calculated for primary charged particles with transverse momenta $p_\text {T}>0.5$ ${\mathrm{GeV}}$ and pseudorapidities $|\eta | <2.5$. Primary particles are defined as those with a decay distance $c\tau$ of at least 10 mm, either stemming from the primary proton–proton interactions or from the decays of shorter-lived particles from the primary proton–proton interactions.

Distributions $f_{{\mathcal {O}}}={1}/{N_\text {ev}} \cdot {\text {d} N}/{\text {d} {\mathcal {O}}}$ were measured for all selected events, $N_\text {ev}$, for the following observables ${\mathcal {O}}$:

- The charged-particle multiplicity, $N_{\mathrm{ch}}$.
- The scalar sum of transverse momenta of selected charged particles, $\sum _{i} p_{\text {T},i} = \sum p_{\text {T}}$.
- The beam thrust, ${\mathcal {B}}$, as proposed in Refs. [20–22]. This is similar to $\sum p_{\text {T}}$ except that in the sum over all charged particles the transverse momentum of each particle is weighted by a factor depending on its pseudorapidity, $\eta$:
  1 $$\begin{aligned} {\mathcal {B}} = \sum _i p_{\text {T},i} \cdot {\text{ e }}^{\, -|\eta _{i}|}. \end{aligned}$$
  As a result, contributions from particles in the forward and backward direction (large values of $|\eta |$) are suppressed with respect to particles emitted at central pseudorapidities ($\eta \approx 0$). The $\sum p_{\text {T}}$ and ${\mathcal {B}}$ observables have different sensitivities to hadronic activity from initial-state radiation.
- The transverse thrust, ${\mathcal {T}}$, as proposed in Ref. [23]:
  2 $$\begin{aligned} {\mathcal {T}}= \max _{{\vec {n}}_{\text{ T }}}\frac{\sum _{i} \left| {\vec {p}}_{\text {T},i} \cdot {\vec {n}}_{\text{ T }}\right| }{\sum _{i} p_{\text {T},i}} \end{aligned}$$
  where the sum runs over all charged particles, and the thrust axis, ${\vec {n}}_{\text{ T }}$, maximises the expression. The solution for ${\vec {n}}_{\text{ T }}$ is found iteratively following the algorithm described in Ref. [24] where one starts with a direction ${\vec {n}}_{\text{ T }}^{(0)}$ and obtains the $j+1$ iteration as
  3 $$\begin{aligned} {\vec {n}}_{\text{ T }}^{(j+1)}= \frac{\sum _{i} \epsilon \left( {\vec {n}}_{\text{ T }}^{(j)} \cdot {\vec {p}}_{\text {T},i} \right) {\vec {p}}_{\text {T},i}}{\left| \sum _{i} \epsilon \left( {\vec {n}}_{\text{ T }}^{(j)} \cdot {\vec {p}}_{\text {T},i} \right) {\vec {p}}_{\text {T},i} \right| } \end{aligned}$$
  where $\epsilon (x)=1$ for $x>0$ and $\epsilon (x)=-1$ for $x<0$.
- The spherocity, ${\mathcal {S}}$, as proposed in Ref. [23]:
  4 $$\begin{aligned} {\mathcal {S}}=\frac{\pi ^2}{4}\underset{{\vec {n}}=(n_x, n_y,0)^\top }{\min } \left( \frac{\sum _{i} \left| {\vec {p}}_{\text {T},i}\times {\vec {n}}\right| }{\sum _{i} p_{\text {T},i}} \right) ^2 \end{aligned}$$
  where the sum runs over all charged particles and the vector ${\vec {n}}$ minimises the expression. In contrast to the closely related sphericity observable [23, 25], which is computed via a tensor diagonalisation, spherocity is simple to calculate since ${\vec {n}}$ always coincides with one of the transverse momentum vectors ${\vec {p}}_{\text {T},i}$ [23].
- The ${\mathcal {F}}$-parameter defined as the ratio of the smaller and larger eigenvalues, $\lambda _1$ and $\lambda _2$,
  5 $$\begin{aligned} {\mathcal {F}}=\frac{\lambda _{1}}{\lambda _{2}} \end{aligned}$$
  of the transverse momentum tensor
  6 $$\begin{aligned} M^\text {lin} = \sum _i\frac{1}{p_{\text {T},i}} \left( \begin{matrix} p_{x,i}^2 &{}\quad p_{x,i}p_{y,i} \\ p_{x,i}p_{y,i} &{}\quad p_{y,i}^2 \end{matrix}\right) \end{aligned}$$
  where the sum runs over the charged particles in an event.

Pencil-like events, e.g. containing two partons emitted in opposite directions in the transverse plane, are characterised by values of ${\mathcal {S}}$, ${\mathcal {T}}$, and ${\mathcal {F}}$ close to 0, 1, and 0 respectively. The corresponding values of these observables for spherical events, e.g. containing several partons emitted isotropically, are close to 1, $2/\pi$, and 1 respectively. While the event-shape observables ${\mathcal {S}}$, ${\mathcal {T}}$, and ${\mathcal {F}}$ show very high correlations among themselves, they are weakly correlated with $N_{\mathrm{ch}}$, $\sum p_{\text {T}}$, and beam thrust.

The observables were calculated after removing the *Z*-boson decay products. The fiducial *Z*-boson phase-space region requires a decay into a pair of oppositely charged leptons, either electrons or muons,^3^ where each lepton must have $p_\text {T} > 20$ GeV and $|\eta | < 2.4$, with a lepton–antilepton invariant mass in the interval [66, 116] GeV. This mass window contains the *Z*-resonance peak and is wide enough to allow the multijet background to be determined from the sideband regions.

Each observable was determined in the following ranges of the transverse momentum of the *Z* boson, $p_\text {T}(\ell ^{+}\ell ^{-})$, calculated from the four-momenta of the lepton and antilepton: 0–6, 6–12, 12–25, and ${\ge } 25$ ${\mathrm{GeV}}$. As mentioned in Sect. 1, events at small $p_\text {T}(\ell ^{+}\ell ^{-})$ are expected to be particularly sensitive to the UE activity, while events with large $p_\text {T}(\ell ^{+}\ell ^{-})$ values (${\ge } 25$ ${\mathrm{GeV}}$) are expected to contain significant contributions from jet production coming from the hard scattering process. The lowest $p_\text {T}(\ell ^{+}\ell ^{-})$ range (0–6 ${\mathrm{GeV}}$) was chosen accordingly as a compromise between small bin size and minimising migration effects. The ranges at higher $p_\text {T}(\ell ^{+}\ell ^{-})$ were each defined so as to contain about the same number of events as the 0–6 ${\mathrm{GeV}}$ range.

In simulated events, particle-level leptons are defined as so-called dressed leptons, obtained by adding to the stable lepton four-momentum the four-momenta of any photons within a cone of ${\Delta R}_{\ell ,\gamma } = 0.1$ [26] and which do not stem from hadron or $\tau$ decays.

## ATLAS detector

The ATLAS detector, described in detail in Ref. [18], covers almost the full solid angle around the collision point. The components relevant to this analysis are the tracking detectors, the liquid-argon (LAr) electromagnetic sampling calorimeters (ECAL) and the muon spectrometer (MS).

The inner tracking detector (ID), consisting of a silicon pixel detector (pixel), a silicon microstrip tracker (SCT) and a straw-tube transition radiation tracker (TRT), covers the full azimuthal angle $\phi$ and the pseudorapidity range $|\eta | \le 2.5$. These individual tracking detectors are placed from inside to outside at a radial distance *r* from the beam line of 50.5–150, 299–560 and 563–1066 mm respectively, within a 2 T axial magnetic field generated by a solenoid surrounding the ID. The inner detector barrel (end-caps) consists of 3 ($2 \times 3$) pixel layers, 4 ($2 \times 9$) layers of double-sided SCT silicon microstrip modules, and 73 ($2 \times 160$) layers of TRT straw-tubes. The typical position resolutions of these subdetectors are 10, 17 and $130~{\upmu \mathrm{m}}$ respectively for the *r*–$\phi$ coordinates. The pixel and SCT detectors provide *r*–*z* coordinate measurements with typical resolutions of 115 and $580~{\upmu \mathrm{m}}$ respectively. The TRT covers $|\eta | \le 2.0$. A charged particle traversing the barrel part of the ID leads typically to 11 silicon hits (3 pixel clusters and 8 microstrip clusters) and more than 30 straw-tube hits.

A high-granularity lead/liquid-argon electromagnetic sampling calorimeter [27] covers the pseudorapidity range $|\eta | \le 3.2$. Hadronic calorimetry in the range $|\eta | \le 1.7$ is provided by an iron/scintillator-tile calorimeter, consisting of a central barrel and two smaller extended barrel cylinders, one on either side of the central barrel. In the end-caps ($|\eta | \ge 1.5$), the acceptance of the LAr hadronic calorimeters matches the outer $|\eta |$ limits of the end-cap electromagnetic calorimeters. The LAr forward calorimeters provide electromagnetic and hadronic energy measurements, and extend the coverage to $|\eta | \le 4.9$.

The muon spectrometer measures the deflection of muons in large superconducting air-core toroid magnets in the pseudorapidity range $|\eta | \le 2.7$. It is instrumented with separate trigger and high-precision tracking chambers. Over most of the $\eta$ range, a precision measurement of the track coordinates is provided by monitored drift tubes. Cathode strip chambers with higher granularity are used in the innermost plane over the range $2.0 \le |\eta | \le 2.7$, where particle fluxes are higher.

The trigger system utilises two stages: a hardware-based Level-1 trigger followed by a software-based high-level trigger, consisting of the Level-2 and Event Filter [28] stages. In the Level-1 trigger, electron candidates are selected by requiring that the signal in adjacent electromagnetic calorimeter trigger towers exceed a certain transverse energy, $E_{\text{ T }}$, threshold, depending on the detector $\eta$. The Event Filter uses the offline reconstruction and identification algorithms to apply the final electron selection in the trigger. The $Z \rightarrow e^{+}e^{-}$ events were selected in this analysis by using a dielectron trigger in the region $|\eta | \le 2.5$ with an electron transverse energy threshold of 12 GeV for each electron.

The muon trigger system, which covers the pseudorapidity range $|\eta | \le 2.4$, uses the signals of resistive-plate chambers in the barrel ($|\eta |<1.05$) and thin-gap chambers in the end-cap regions ($1.05< |\eta | < 2.4$). The $Z \rightarrow \mu ^{+}\mu ^{-}$ events in this analysis were selected with a trigger that requires the presence of at least one muon candidate reconstructed in the muon spectrometer with transverse momentum of at least 11 GeV at Level-1 and 18 GeV at the Event Filter stage.

## Monte Carlo simulations

Monte Carlo simulated samples for the signal and the various background processes were generated at particle level before being passed through a Geant4-based [29] simulation of the ATLAS detector response  [30] followed by the detector reconstruction. These samples were used to correct the measured observables for detector effects and to estimate related systematic uncertainties.

The signal process was simulated with two different event generators in order to quantify the model uncertainty in the correction of the measured distributions to particle level: the leading-order (LO) generator Pythia 8.150 using the CTEQ6L1 [31] parton distribution functions (PDFs), and the LO generator Sherpa 1.3.1 using the CT10 next-to-leading-order (NLO) PDF set [32].

For the Pythia 8 samples, inclusively produced $Z \rightarrow \ell ^{+} \ell ^{-}$ events were generated. The Pythia 8 generator uses a leading-logarithm $p_\text {T}$-ordered parton shower (PS) model which is matched to LO matrix element calculations. Multiple partonic interactions are phenomenologically modelled by perturbative QCD parton–parton scattering processes down to an effective $p_\text {T}$ threshold (Sjöstrand–van Zijl model [33]) accompanied by the mechanism of colour reconnection of colour strings. The phenomenological description of hadronisation is implemented using the Lund string model [34]. The Pythia 8 samples were generated with model parameters tuned to Tevatron and earlier LHC data (4C tune [35]).

For the Sherpa signal samples, tree-level matrix elements for $pp \rightarrow Z + X, Z \rightarrow \ell ^{+} \ell ^{-}$ were used with up to five additional final-state partons. The model used for MPI in Sherpa is also based on the Sjöstrand–van Zijl model, but the mechanism of colour reconnection is not activated. Hadronisation modelling uses a cluster hadronisation scheme.

The background processes ($t\bar{t}$, $Z \rightarrow \tau ^{+} \tau ^{-}$, *ZZ*, and *WZ* production) relevant to the analysis were generated with Sherpa version 1.4.0 in the case of $Z \rightarrow \tau ^{+} \tau ^{-}$, *ZZ*, and *WZ* production, and with version 1.3.1 in the case of $t\bar{t}$ production using in both cases the CT10 NLO PDF set. The default parameter tuning performed by the Sherpa authors was used.

The events of the MC signal samples were generated with and without overlaid simulated pile-up events in order to validate the data-driven PU correction method with simulated events. The Pythia 8 generator (version 8.150 with the CTEQ6L1 [31] PDF and 4C tune) was used to simulate the pile-up events. The number of PU events overlaid was chosen to reproduce the average number of proton–proton collisions per bunch crossing observed in the data analysed.

For comparison with corrected distributions, three different, recent versions of MC event generators were used to provide predictions for the signal at particle level: Sherpa 2.2.0 with up to two additional partons at NLO and with three additional partons at LO and taking the NLO matrix element calculations for virtual contributions from OpenLoops [36] with the NNPDF 3.0 NNLO PDF set [37]; Pythia 8.212 with LO matrix element calculations using the NNPDF2.3 LO PDF set [38]; and Herwig 7.0 [1] taking the NLO matrix element calculations for real emissions from MadGraph [39] and for virtual contributions from OpenLoops using the MMHT2014 PDF set [40]. The Herwig 7 event generator implements a cluster hadronisation scheme with parton showering ordered by emission angle. All the parameters relevant to the UE modelling were set to values chosen by the corresponding MC generator authors: while these were the default values in Sherpa and Herwig 7, for Pythia 8 the Monash 2013 tune to LHC data was chosen for the settings of the UE parameters [41]. The A14 Pythia 8 tune of the ATLAS collaboration [42] gives predictions for the event-shape observables which are very close to, and differ by at most $5~\%$ from, the ones obtained by the Monash 2013 tune.

The treatment of QED radiation is generator-specific and modelled differently in Pythia 8 compared to Sherpa and Herwig 7. The latter radiate more soft-collinear and wide-angle photons than Pythia 8, as a result of their usage of a YFS-based model [43] for QED emissions.

## Analysis

Since the track-based observables are sensitive to pile-up effects, the analysis was restricted to a subsample of $1.1~{\text {fb}}^{-1}$ integrated luminosity of the 2011 dataset, in which the mean number of *pp* collisions per bunch crossing was typically only around five and not larger than seven. With this dataset the results are in most cases already dominated by systematic uncertainties. After the event and track selection the event-shape observables were corrected first for PU and then for background contributions, and finally corrected for detector effects.

### Event selection

Only events containing a “primary vertex” (PV) as defined below were processed, to reject events from cosmic-ray muons and other non-collision background. A reconstructed vertex must have at least one track with a minimum $p_\text {T}^\text {trk}$ of 400 MeV from the region inside the detector where the collisions take place. The PV is defined as the vertex with the highest $\sum ({p_\text {T}^\text {trk}})^2$ value of tracks associated with the vertex.

Selected electrons and muons were required to have a $p_{\text {T}}$ of at least 20 ${\mathrm{GeV}}$ and a pseudorapidity $|\eta | < 2.4$. In the case of electrons, the $\eta$ range $1.37< |\eta | < 1.52$ was excluded in order to avoid large amounts of passive detector material in the region between the barrel and end-cap ECAL. Electron candidates were identified using information from the shower shape in the ECAL, from the association between ID tracks and ECAL energy clusters, and from the number of transition radiation hits in the TRT [44]. Muon candidates were built from track segments in the MS matched to tracks in the ID [45]. Electron candidates were required to have a transverse impact parameter with respect to the PV of $|d_0| < 5$ mm and muon candidates of $|d_0| < 3 \times \sigma _{d_0}$, with $\sigma _{d_0}$ being the transverse impact parameter resolution of the muon candidate. In addition, muon candidates had to pass the longitudinal impact parameter requirement $|z_0| < 10$ mm. While no isolation criterion was required for muon candidates, the selection requirements for electron candidates contain implicitly some isolation cuts. Only events containing exactly one pair of oppositely charged leptons passing the selection cuts as described above were considered. These were treated as $Z \rightarrow \ell ^{+} \ell ^{-}$ signal events if the $\ell ^{+}\ell ^{-}$ invariant mass was in the region $m_{\ell ^{+}\ell ^{-}} \in [66,116]$ ${\mathrm{GeV}}$. After all selection requirements, about $2.6 \times 10^{5}$ electron–positron events (“electron channel analysis”) and $4.1 \times 10^{5}$ muon–antimuon events (“muon channel analysis”) remained.

### Track selection

To calculate the event-shape observables for charged particles, tracks fulfilling the following criteria, identical to those used in Ref. [46], were selected:

1. at least one hit in the pixel subdetector;
2. a hit in the innermost pixel layer if the reconstructed trajectory traversed an active pixel module;
3. at least six SCT hits;
4. the transverse momentum of the track $p_\text {T}^\text {trk} > 0.5$ ${\mathrm{GeV}}$;
5. the pseudorapidity of the track $|\eta ^\text {trk}| < 2.5$;
6. the transverse impact parameter of the track with respect to the PV $|d_0| < 1.5$ mm;
7. the longitudinal impact parameter of the track with respect to the PV $|z_0| \sin \theta < 1.5$ mm;
8. a goodness-of-fit probability greater than 0.01 for tracks with $p_\text {T}^\text {trk} > 10$ ${\mathrm{GeV}}$.

The first two requirements greatly reduce the number of tracks from non-primary particles, which are those originating from particle decays and interactions with material in the inner detector. The third one imposes an indirect constraint on the minimum track length and hence on the precision of the track parameters. The kinematic requirements (4. and 5.) imposed on the track selection are driven by the $\eta$-acceptance of the inner detector and the need for an approximately constant reconstruction efficiency as a function of $p_\text {T}^\text {trk}$. The impact parameter requirements (6. and 7.) aim to suppress tracks not originating from the PV of the event. The cut on the goodness-of-fit probability reduces the fraction of mismeasured tracks at high $p_\text {T}^\text {trk}$ values. With these requirements except for 4., the track reconstruction efficiency rises in the $|\eta ^\text {trk}|<1.0$ range from $80~\%$ at $p_\text {T}^\text {trk}=$ 400 MeV to around $90~\%$ at $p_\text {T}^\text {trk}=$ 5 GeV and then stays constant. For higher $|\eta ^\text {trk}|$ values the efficiency variation is stronger: at $|\eta ^\text {trk}|=2.5$ the efficiency rises from around $50~\%$ at $p_\text {T}^\text {trk}= 400$ MeV to around $80~\%$ at 5 GeV.

### Lepton track removal

Since this analysis aims to measure charged-particle distributions, the decay products of the *Z*-boson were removed from the set of tracks used to calculate the observables. Electrons can interact with the material in front of the ECAL leading to multiple tracks as a result of bremsstrahlung and photon conversion. Hence, tracks were not used in the calculation of each event-shape variable if they fell inside a cone of ${\Delta R}_{e,\mathrm {trk}} = 0.1$ around any selected electron or positron. In order to treat the electron and muon channel analyses as similarly as possible, this approach was also applied to the muon channel. It was checked that the observables changed in data and in simulated signal samples in the same way within statistical uncertainties when the cone size was varied within a factor of two.

### Pile-up correction

If another proton–proton interaction is spatially close to the primary interaction where the *Z*-boson is produced, it is possible that the vertex algorithm assigns tracks from the PU interaction to the reconstructed primary vertex. The PU correction used in this analysis is based on the “Hit Backspace Once More” (HBOM) approach [47], which relies on recursively applying a smearing effect to a measured distribution, in this case the effect from the contamination by tracks selected from pile-up. An event-shape distribution without pile-up tracks, $f_{{\mathcal {O}}}^{0}$, is changed to an event-shape distribution, $f_{{\mathcal {O}}}^{1}$, when pile-up tracks that are passing the selection cuts are taken into account in the calculation of the event-shape observables. By adding once more pile-up tracks one obtains a distribution, $f_{{\mathcal {O}}}^{2}$. This procedure can be repeated *k* times, resulting in the distribution $f_{{\mathcal {O}}}^{k}$. Knowing $f_{{\mathcal {O}}}^{k}$ as a function of *k* allows one to extrapolate from the PU-contaminated distribution $f_{{\mathcal {O}}}^{k=1}$ to $f_{{\mathcal {O}}}^{k=0}$, hence to the distribution without PU contamination. In the analysis, the *k*-th application of the PU effect on an event-shape observable was parameterised by an *n*th-order polynomial function, *P*(*k*), in the following called HBOM parametrisation. The procedure was carried out in each individual bin of the event-shape observables using the Professor toolkit [48] to determine the parameters of *P*(*k*) by means of a singular value decomposition [49].

The PU effect on the observables was estimated by constructing a library of “pseudo-vertices” containing tracks passing the track selection requirements with respect to vertices that are well isolated from the PV and any other vertex (see Sect. 5.1). Typically, these vertices originate from PU and are therefore called PU vertices in the following. In addition to the track parameters, the library also stores the position of the corresponding PU vertex along the beam-line, $z_\text {vtx}^{\text {PU}}$. All vertices of events passing the nominal event selection were potential candidates for the library. However, to safeguard against cases in which a single vertex is falsely reconstructed as two or more vertices close in *z* (“split vertices”) it was required that the selected vertices have a minimum distance along the beam line from any other vertex, $\Delta z_\text {min}^\text {vtx}$, of 60 mm. In the process of building a pseudo-vertex at $z_\text {vtx}^{\text {PU}}$, tracks were required to satisfy

7 $$\begin{aligned} \left| \left( z_\text {vtx}^{\mathrm{PU}} - z_{0,\text {trk}} \right) \, \sin \theta _\text {trk} \right| < 3~\text {mm}. \end{aligned}$$

This selection window is larger than the nominal track selection window with respect to the PV in order to account for the possibility that the PV marginally overlaps with a pseudo-vertex. Parameters of each track fulfilling the requirements above were stored to form the pseudo-vertex.

The effect of the pile-up contamination was then quantified as follows:

1. For each event, draw a random number, $N_\text {rdm}$, from the distribution of the number of vertices per event.
2. Obtain $N_\text {rdm}$ random vertex positions, $z_{\text {rdm}, i}$ ($i=1,\ldots , N_\text {rdm}$), from the distribution of reconstructed pile-up vertices fulfilling the $\Delta z_\text {min}^\text {vtx}$ requirement, and for each of those, a random pseudo-vertex from the library entry corresponding to $z_{\text {rdm}, i}$, each containing an independent number of tracks.
3. Any track *j* belonging to such a selected pseudo-vertex *i* with a longitudinal impact parameter with respect to the pseudo-vertex $z_{0,ij}^{\mathrm{PU}} \, \sin \theta _\text {trk}^{ij}$ is then added to the list of an event’s signal tracks if it falls in the signal track selection window
  8 $$\begin{aligned} \left| \,\, \left( z_{\text {rdm}, i} + {z_{0,ij}^{\mathrm{PU}}} - z_\text {PV} \right) \, \sin \theta _\text {trk}^{ij} \,\, \right| < 1.5~\text {mm}. \end{aligned}$$

With these additional tracks each observable was then re-calculated to determine $f_{{\mathcal {O}}}^{k}$ for $k=2,\ldots , 11$. The HBOM parameterisation for $f_{{\mathcal {O}}}^{k}$ as a function of *k* was parameterised by a third-order polynomial used to extrapolate to $k=0$.

The PU correction varies when changing the random seed of the selection. To reflect the statistical nature of the PU correction, ten different statistically independent versions of the PU correction were determined. The final PU correction was the mean of these ten PU corrections.

Using a library of pseudo-vertices built from detector-simulated PU events (see Sect. 4), four tests were performed to validate the PU correction method.

1. In the first “forward-closure” test, the effect of PU contamination in the event-shape observables as modelled by the HBOM parameterisation was applied to a simulated sample without PU events overlaid by adding to each event-shape observable $f_{{\mathcal {O}}}$ binwise the term $P(1)-P(0)$. It was found that event-shape observables obtained in this way were in very good agreement with those obtained where PU events were overlaid. Only in the charged-multiplicity bin $N_{\mathrm {trk}}=0$ was a sizeable non-closure of the order of 10 % (22 %) to 20 % (34 %) in the muon (electron) channel observed. This effect is likely caused by an unavoidable bias in the vertex selection for the PU library and was considered as a systematic uncertainty.
2. In the second “backward-closure” test, the HBOM parameterisation was used to correct event-shape observables in simulated samples containing PU events to distributions without PU effect. The results were found to be in very good agreement with the corresponding samples without PU events overlaid. As in the “forward-closure” test, the only non-closure was observed in the charged-multiplicity bin $N_{\mathrm {trk}}=0$.
3. In the third test, the selection cuts defining the PU library were varied and no significant deviations beyond the systematic uncertainties assigned to the HBOM method were observed.
4. The $z_{\text {rdm}}$ distribution of the pseudo-vertices in the library is similar but not identical to the $z^{\mathrm{PU}}_\text {vtx}$ distribution of all PU vertices. In the fourth test, the $z^{\mathrm{PU}}_\text {vtx}$ distribution was used instead of the $z_{\text {rdm}}$ distribution and again the PU-corrected result was found to be in very good agreement with the corresponding samples without PU events overlaid.

While for $N_{\mathrm{ch}}$ the PU correction varied from $20~\%$ at low multiplicities to $40~\%$ at high multiplicities, the PU corrections for all other event-shape observables were at most 15–$20~\%$ for both the electron and the muon channel.

### Background treatment

In addition to $Z \rightarrow \ell ^{+} \ell ^{-}$ events the following background sources were assumed to contribute to the signal region: events from multijet production with misidentified lepton candidates or leptons from decays of hadrons, production of $t \bar{t}$ quark pairs, production of *Z* bosons decaying into a $\tau ^{+} \tau ^{-}$ pair with subsequent decays to electrons or muons, and diboson production *ZZ* and *WZ* with gauge-boson decays into leptons.

All background contributions were found to be small compared to the number of $Z \rightarrow \ell ^{+} \ell ^{-}$ events, with the most prominent contribution coming from multijet events. While the effect of multijet events was estimated from data and corrected for, no explicit correction was made for the other background sources because their contribution was found to be very small: using MC simulation the background fraction from $t \bar{t}$, $Z \rightarrow \tau ^{+} \tau ^{-}$, and diboson production *WZ* and *ZZ* was estimated to be about $0.25~\%$ for the complete *Z*-boson transverse momentum phase space. About $70~\%$ of these background contributions (*ZZ* production as well as $Z\rightarrow \tau ^{+}\tau ^{-}$ events) had event-shape distributions very similar to the ones of the signal process. The fraction of $t \bar{t}$ (*WZ*) background, showing significantly different event-shape distributions in the MC simulation compared to the signal process, was found to be 0.04–$0.05~\%$ ($0.03~\%$) in the full $p_\text {T}(\ell ^{+}\ell ^{-})$ spectrum. Since these background fractions are very small and other systematic uncertainties significantly larger, no correction for $t \bar{t}$ and *WZ* background was applied.

In both lepton channels, the relative number of multijet events as well as their event-shape observables were estimated from data as described below. The measured, PU-corrected event-shape observables $f_{{\mathcal {O}}}^\text {meas}$ were then corrected by applying bin-wise the multiplicative factor $1-{f_{{\mathcal {O}}}^\text {multijet}}/{f_{{\mathcal {O}}}^\text {meas}}$ where $f_{{\mathcal {O}}}^\text {multijet}$ represents the estimate of the event-shape observable for multijet events.

Modified event and/or lepton selections for the electron and muon channels, as described below, were performed to obtain the dilepton invariant mass distributions, $m_{\ell \ell }^\text {multijet}$, dominated by contributions from multijet events. These distributions were fitted using a linear function, $g^\text {multijet}(m_{\ell \ell })$, omitting the peak region $m_{\ell \ell }^\text {multijet}\in [77,97]$ ${\mathrm{GeV}}$ to avoid a fit bias from remaining peaking signal contributions. Assuming that only multijet events contribute to these samples, the integral, $I^\text {multijet}$, of the fit function over the whole signal window ($m_{\ell \ell }^\text {multijet}\in [66,116]$ ${\mathrm{GeV}}$) was used to estimate the amount of multijet background entering the signal region. The event-shape distributions obtained with the modified selection criteria were used as an estimate of the corresponding multijet background shape and were then scaled so as to match the total amount of the multijet background, $I^\text {multijet}$. This procedure was performed for all $p_\text {T}(\ell ^{+}\ell ^{-})$ ranges separately since the amount of the multijet background depends on $p_\text {T}(\ell ^{+}\ell ^{-})$ and rises with increasing $p_\text {T}(\ell ^{+}\ell ^{-})$. For the fully inclusive distributions, it amounted to $0.7~\%$ in the electron channel and to $1.9~\%$ in the muon channel.

In the electron case, two different samples with either different event selection criteria or different lepton selection criteria were considered in estimating the number of multijet events and the distributions of their event-shape observables. In the first sample, the lepton-pair selection was changed from opposite-sign to same-sign charged electrons (i.e. an electron–electron or positron–positron pair). Drell–Yan contributions to this multijet-enriched sample were estimated to be of the order $15~\%$. This sample was used to estimate the number of multijet events and their event-shape observables as described above, assuming the same selection efficiency for multijet events in the opposite-sign and same-sign electrons selection. In addition, opposite-sign and same-sign electron events were selected with significantly looser electron selection requirements to obtain a second multijet-enriched sample. With the second sample, it was verified that the opposite-sign and same-sign requirements select nearly equal numbers of multijet events and that the event-shape distributions for multijet background agree for the opposite-sign and same-sign electron selections. The multijet background correction factors for the electron channel were found to be very close to one, where the largest change in the event-shape observables was not more than $3~\%$.

In the muon case, an isolation criterion, which is based on the scalar sum of transverse momenta of tracks found in a cone in $\eta$–$\phi$ space around the muon, was introduced to obtain a sample with a much smaller multijet background contribution. The fraction of multijet background was then determined by subtracting the $m_{\mu \mu }$ distribution for the isolated muon selection, assuming negligible contributions from multijet events, from the one for the standard muon selection, since the two have very similar $Z \rightarrow \mu ^{+}\mu ^{-}$ selection efficiencies. Contributions from signal events to this multijet-dominated distribution were estimated to be of the order of $5~\%$. The event-shape distributions of multijet background were estimated accordingly by subtracting the event-shape distributions for the isolated muon selection from the one of the standard selection. Compared to the electron channel, the multijet background correction factors in the muon channel were found to deviate significantly more from one and to show more functional dependence in the event-shape distributions.

As a cross-check of the background subtraction procedure the reconstructed event-shape distributions were measured for smaller $m_{\ell \ell }$ signal window widths of 30, 20, and 10 GeV while using the background estimate from the standard $m_{\ell \ell }$ selection applied to the narrower $m_{\ell \ell }$ signal window. By narrowing the $m_{\ell \ell }$ window, the signal-to-background ratio is increased and as a result the effect from background becomes smaller. Differences seen in some individual bins were found to be much smaller than the systematic uncertainties, and no systematic dependence of the event-shape distributions as a function of the $m_{\ell \ell }$ window size was observed.

### Unfolding

The observables were measured in different $p_\text {T}(\ell ^{+}\ell ^{-})$ ranges and corrected for contributions from non-primary particles, detector efficiency and resolution effects using an unfolding technique.

The bin sizes for the distributions of the event-shape observables were chosen taking into account two aspects: to have a fine enough binning to best see the shape of each distribution, and to have enough events in each bin, particularly in the tails of the distributions. It was explicitly checked with unfolding closure tests as described below that the bin sizes were not too small compared to the experimental resolution.

For the unfolding of the measured observables a Bayesian approach was applied [50]. The unfolding procedure requires an input distribution (called the prior distribution), which was taken from MC signal samples, and the detector response matrix $M_{ij}$. The matrix, $M_{ij}$, determined using simulated signal samples, quantifies the probability that an event with the event-generator value (at particle level) in bin *i* of a distribution is reconstructed in bin *j*. Since the unfolding result depends on the prior distribution, the Bayesian unfolding is performed in an iterative way until convergence, minimising the dependence on the prior distribution. For the iterative Bayesian unfolding the Imagiro framework [51] was used, with improvements, as proposed in Ref. [52], to the error calculation in the original work described in Ref. [50]. The number of iteration steps in the Imagiro framework is obtained in an automatised way. Distributions of Pythia 8 events at reconstruction level were unfolded with a detector response matrix obtained with simulated Sherpa events and vice versa. The level of agreement of the unfolded distributions with the particle distributions of the corresponding event generator was quantified by a $\chi ^2$ test and a Kolmogorov–Smirnov (KS) test. The optimal number of iteration steps was set to the number of iteration steps for which the minimum (maximum) of the $\chi ^2$ (KS) test statistic was observed in the simulation. In general, the optimal number of iteration steps was found to be two, except for $\sum p_\text {T}$ in the $p_\text {T}(\ell ^{+}\ell ^{-})$ bin 12–25 ${\mathrm{GeV}}$, in which case it was three.

Since corrections were made for the effect of pile-up on the observables before unfolding, the simulated signal samples used for the prior distribution and the detector response matrix did not contain pile-up events. Signal samples generated with either Pythia 8 or with Sherpa were used to determine the prior distribution and the detector response matrix. The results of the unfolding obtained with these two simulations were then averaged.

The complete analysis chain was tested on reconstructed MC signal samples simulated with either Pythia 8 or Sherpa with overlaid pile-up events generated by Pythia 8. The event-shape observables were corrected for pile-up using the same strategy as in data. The resulting distributions were then unfolded using detector response matrices and priors obtained from the MC signal samples without pile-up. In general, the unfolding results showed good closure: the corrected MC distributions were found to be in very good agreement with the particle-level distributions. This was also the case when events generated by Pythia 8 were unfolded with Sherpa prior distributions and Sherpa detector response matrices and vice versa.

### Systematic uncertainties

Several categories of systematic uncertainties that influence the distributions after corrections and unfolding were quantified.

- **Lepton selection:** Uncertainties in the lepton selection affect not only the selected events but also the reconstructed $p_\text {T}(\ell ^{+}\ell ^{-})$ in data and simulation, and hence are important for the unfolding where the subdivision of the data into different $p_\text {T}(\ell ^{+}\ell ^{-})$ ranges is performed. Variations were performed for each source of systematic uncertainty and were propagated through the unfolding to estimate their effect on the results. For the electron channel, systematic uncertainties in the energy resolution, the energy scale, and the trigger, reconstruction and identification efficiencies were quantified [44, 53]. The largest effect on the event-shape observables was observed from the electron energy scale systematic uncertainties. The total effect was typically in the subpercent range and therefore much smaller than the statistical and other systematic uncertainties. For the muon channel, systematic uncertainties in the observables from the efficiencies (reconstruction and trigger) as well as from the calibration of the reconstructed muon transverse momentum [45] were also typically below the percent level.
- **Track reconstruction:** In order to estimate the effect of uncertainty in the track reconstruction efficiency on the observables, the data distributions were unfolded with a modified detector response matrix taking into account variations of the track reconstruction efficiencies. The relative track reconstruction efficiency systematic uncertainties were estimated as a function of $p_{\text {T}} ^{\text {trk}}$ and $|\eta ^{\text {trk}}|$:
  - For tracks with $|\eta ^{\text {trk}}|<2.1$ the relative uncertainty was estimated to be $1.5~\%$ for tracks with $p_\text {T}^{\text {trk}}$ in the range 500–800 ${\mathrm{MeV}}$ and $0.7~\%$ for all tracks with $p_\text {T}^{\text {trk}} >800$ ${\mathrm{MeV}}$ [46].
  - For tracks with $|\eta ^{\text {trk}}| \ge 2.1$ several effects were assessed to quantify the systematic uncertainty [54]: uncertainties in the modelling of the detector material in particular in the vicinity of service structures and cooling pipes (4–7 %), systematic uncertainties in the track selection related to the requirements on the transverse impact parameter and on the innermost pixel layer to suppress charged particles stemming from interactions with the detector material (1 %), the fraction of mismeasured tracks for transverse momenta above 10 GeV (1.2 % between 10 and 15 GeV, up to 80 % above 30 GeV at high $|\eta ^{\text {trk}}|$ values), and the systematic uncertainty due to the goodness-of-fit probability cut to reduce mismeasured tracks above 10 GeV (10 %).
  The systematic uncertainty in the track reconstruction efficiency was generally found to be the dominant systematic uncertainty for observables where the number of charged particles does not cancel in the definition ($N_{\mathrm{ch}}$, $\sum p_\text {T}$, beam thrust) and reached as high as 10 %. For all other observables, it was typically between 1 and 3 %. The contribution was of the same order when comparing unfolded distributions from the electron channel and the muon channel.
- **Non-primary particles:** The effect from non-primary particles, which are those originating from decays and interactions with material in the inner detector, was taken into account by the unfolding procedure. The fraction and composition of non-primary particles in data is not perfectly modelled by the MC simulation, which is able to reproduce the fraction in data to an accuracy of about 10–$20~\%$ as a result of a fit to the $d_{0}$ distribution [13]. To estimate the corresponding systematic uncertainty, the requirement on the track impact parameter $|d_{0}|$ was varied from the nominal value of 1.5 mm downward to 1.0 mm and upward to 2.5 mm, resulting in a 0.5–4 % change in the fraction of the non-primary particles [13]. The resulting event-shape distributions were unfolded using MC signal samples selected with the same impact parameter requirements to test the stability of the unfolding result. The maximum residual difference was taken as the systematic uncertainty from the impact parameter requirement. The typical relative uncertainty was $2~\%$ or smaller, except for a few individual bins.
- **Pile-up correction:** The standard deviation of the mean PU correction obtained from the ten independent PU corrections was considered as a systematic uncertainty of statistical nature. The default HBOM parameterisations used third-order polynomials giving a very good description of the pile-up effect. Similarly good descriptions were obtained by fourth-order polynomials. The differences between using third-order and fourth-order polynomials were used to quantify the systematic uncertainty coming from the choice of HBOM parameterisation, resulting in systematic uncertainties in the event-shape observables typically below 2 %. In contrast to a $\chi ^2$ fit, the singular value decomposition used to obtain the polynomial parameterisation does not take into account uncertainties. Hence, there is no *a priori* goodness-of-fit measure for the parameterisation. If the polynomial *P*(*k*) provides a good prediction of each HBOM point, $f_{\mathcal O}^{k}$, and if each HBOM point fluctuates around *P*(*k*) with the same uncertainty $\sigma$, then one expects $\sum _{k=1}^{11}(P(k)-f_{\mathcal O}^{k})^2/\sigma ^{2}=11$. This equation was used to estimate the size of such a typical uncertainty $\sigma$ for each bin of the observables. The so-determined average uncertainty was then taken as a systematic uncertainty for the HBOM extrapolation. This systematic uncertainty is similar in size to the variation from third-order to fourth-order polynomials. A further check was made by omitting the *k*-th point when calculating the parameterisations. In each bin, the largest deviation of these extrapolations from the nominal extrapolation was taken as a systematic uncertainty. This deviation was found to rarely exceed $1~\%$ and hence is negligible in most bins. To obtain the total uncertainty of the method, the four systematic uncertainties were added in quadrature. The $N_\text {trk}=0$ bin showed a bias in the MC tests due to the track and vertex selections, leading to a sizeable non-closure for this particular bin. An additional correction for this expected non-closure as determined from simulation was performed and the full size of the correction was applied as an additional uncertainty. The systematic uncertainty in the pile-up correction propagated through the unfolding led to a systematic uncertainty in the event-shape observables of 1 to 3 % with the exception of some bins with few events. In general, fewer events in a given bin corresponded to a larger systematic uncertainty in the PU correction. The PU correction systematic uncertainty was found to have negligible dependence on $p_\text {T}(\ell ^{+}\ell ^{-})$. The results for the electron and muon channel were of comparable magnitude.
- **Multijet background correction:** For the electron channel, a systematic uncertainty was assigned to the shape of the multijet background event-shapes by taking into account the differences between the distributions obtained with the same-sign and opposite-sign events with the loosened electron selection criteria. In order to estimate the systematic uncertainty in the multijet background in the muon channel, the calculation of the multijet background correction factors was repeated for several variations of the isolation criteria. The largest difference per bin from the central isolation was taken as the systematic uncertainty. The systematic uncertainty in the background correction was found to be negligible in almost all bins of all observables. Similar to the pile-up correction systematic uncertainty, significant contributions were observed in bins with few events.
- **Unfolding:** The model uncertainty in the unfolding was estimated by using Pythia 8 and Sherpa separately for the prior distribution and the detector response matrix. The systematic uncertainty corresponding to the unfolding with different priors and detector response matrices was taken from the differences between the central value and the individual results obtained with Pythia 8 and Sherpa. For most observables, the unfolding model error was of the order of 1 % or below, except for poorly populated bins in which it can reach up to 15 %. The sizes observed in the electron and the muon channels were found to be in good agreement.

**Table 1:** Ranges of the relative uncertainties $\frac{\delta _{{\mathcal {O}}}}{{\mathcal {O}}}$ of the event-shape observables ${\mathcal {O}}$ for the electron and muon channels indicated by ($e^{+}e^{-}$) and ($\mu ^{+}\mu ^{-}$) for the $p_\text {T}(\ell ^{+}\ell ^{-})$ range 0–6 ${\mathrm{GeV}}$ in percent. The superscripts denote the statistical (‘stat’) and the individual systematic uncertainties in the lepton reconstruction and identification (‘Lepton’), track reconstruction efficiency (‘Tracking’), non-primary particles (‘Non-prim.’), pile-up correction (‘PU’), multijet background (‘Multijet’), and the unfolding (‘Unfold’)

Observable	Channel	${\delta ^{\mathrm{stat}}_{{\mathcal {O}}}}$ $[\%]$	${\delta ^{\mathrm{Lepton}}_{{\mathcal {O}}}}$ $[\%]$	${\delta ^{\mathrm{Tracking}}_{{\mathcal {O}}}}$ $[\%]$	${\delta ^{\mathrm{Non-Prim.}}_{{\mathcal {O}}}}$ $[\%]$	${\delta ^{\mathrm{PU}}_{{\mathcal {O}}}}$ $[\%]$	${\delta ^{\mathrm{Multijet}}_{{\mathcal {O}}}}$ $[\%]$	${\delta ^{\mathrm{Unfold}}_{{\mathcal {O}}}}$ $[\%]$
$N_{\mathrm{ch}}$	($e^{+}e^{-}$)	1–5	0.2–0.6	${<}0.1$–9	0.1–2.5	0.5–28	${<}0.1$–0.6	0.2–8.4
	($\mu ^{+}\mu ^{-}$)	0.8–4.3	0.1–0.5	0.3–9.9	0.1–2.1	0.2–19	${<}0.1$–0.4	0.1–4.4
$\sum p_{\text {T}}$	($e^{+}e^{-}$)	1–3	0.1–0.5	0.3–5.5	${<}0.1$–1.3	0.13–6.8	0.01–0.4	${<}0.1$–0.8
	($\mu ^{+}\mu ^{-}$)	0.8–2.4	0.1–0.5	0.3–5.3	${<}0.1$–1.3	0.2–3.5	${<}0.1$–0.3	${<}0.1$–1
${\mathcal {B}}$	($e^{+}e^{-}$)	0.8–14	0.1–2.4	${<}0.1$–6.2	0.1–2.1	0.1–36	${<}0.1$–2.1	0.2–2.9
	($\mu ^{+}\mu ^{-}$)	0.6–9.5	0.1–2.0	${<}0.1$–5.8	${<}0.1$–4.5	0.2–14	${<}0.1$–1.6	0.1–5.9
${\mathcal {T}}$	($e^{+}e^{-}$)	0.6–4.4	0.1–0.5	0.2–2.2	0.1–1.6	0.1–4.7	0.1–0.3	0.1–2.6
	($\mu ^{+}\mu ^{-}$)	0.5–3.5	0.1–0.6	0.1–2.0	0.1–1.2	0.1–4.0	${<}0.1$	0.2–2.9
${\mathcal {S}}$	($e^{+}e^{-}$)	0.6–3.8	0.1–0.4	0.3–2.6	0.1–1.4	0.1–4.3	0.1–0.4	0.1–2.2
	($\mu ^{+}\mu ^{-}$)	0.5–3.0	0.1–0.4	0.1–1.9	0.1–1.8	0.1–4.1	${<}0.1$	0.1–5.4
${\mathcal {F}}$	($e^{+}e^{-}$)	0.6–3.6	0.1–0.5	0.3–1.6	0.1–1.5	0.1–1.7	0.1–0.3	0.1–2.0
	($\mu ^{+}\mu ^{-}$)	0.5–2.9	0.1–0.3	0.1–1.9	0.1–1.2	0.1–1.6	${<}0.1$	0.1–1.9

**Table 2:** Ranges of the relative uncertainties $\frac{\delta _{{\mathcal {O}}}}{{\mathcal {O}}}$ of the event-shape observables ${\mathcal {O}}$ for the electron and muon channels indicated by ($e^{+}e^{-}$) and ($\mu ^{+}\mu ^{-}$) for the $p_\text {T}(\ell ^{+}\ell ^{-})$ range 6–12 ${\mathrm{GeV}}$ in percent. The superscripts denote the statistical (‘stat’) and the individual systematic uncertainties in the lepton reconstruction and identification (‘Lepton’), track reconstruction efficiency (‘Tracking’), non-primary particles (‘Non-prim.’), pile-up correction (‘PU’), multijet background (‘Multijet’), and the unfolding (‘Unfold’)

Observable	Channel	${\delta ^{\mathrm{stat}}_{{\mathcal {O}}}}$ $[\%]$	${\delta ^{\mathrm{Lepton}}_{{\mathcal {O}}}}$ $[\%]$	${\delta ^{\mathrm{Tracking}}_{{\mathcal {O}}}}$ $[\%]$	${\delta ^{\mathrm{Non-Prim.}}_{{\mathcal {O}}}}$ $[\%]$	${\delta ^{\mathrm{PU}}_{{\mathcal {O}}}}$ $[\%]$	${\delta ^{\mathrm{Multijet}}_{{\mathcal {O}}}}$ $[\%]$	${\delta ^{\mathrm{Unfold}}_{{\mathcal {O}}}}$ $[\%]$
$N_{\mathrm{ch}}$	($e^{+}e^{-}$)	1–10	0.1–2.2	0.2–10	0.2–6.6	0.1–24	${<}0.1$–0.2	${<}0.1$–10
	($\mu ^{+}\mu ^{-}$)	0.8–8.4	0.1–1.8	${<}0.1$–11.4	0.1–4.5	0.6–21	${<}0.1$–0.4	0.7–7.7
$\sum p_{\text {T}}$	($e^{+}e^{-}$)	1–2.3	0.1–0.5	0.1–5.3	${<}0.1$–1.9	0.4–2.9	${<}0.1$–0.3	${<}0.1$–1.8
	($\mu ^{+}\mu ^{-}$)	0.8–1.8	0.1–0.6	${<}0.1$–4.9	${<}0.1$–1.4	0.1–3.2	${<}0.1$–0.3	0.1–1.7
${\mathcal {B}}$	($e^{+}e^{-}$)	0.7–8.8	0.1–1.5	0.2–4.3	0.1–1.5	${<}0.1$–19	${<}0.1$–1	${<}0.1$–2.4
	($\mu ^{+}\mu ^{-}$)	0.6–6.7	0.1–1	0.3–3.9	${<}0.1$–1.9	0.1–10	${<}0.1$–0.6	0.1–2.4
${\mathcal {T}}$	($e^{+}e^{-}$)	0.6–4.7	0.1–0.5	0.2–2.2	0.1–1.5	0.1–2.9	0.1–0.5	0.1–2.5
	($\mu ^{+}\mu ^{-}$)	0.5–3.7	0.1–1	0.2–2.8	0.1–1	0.1–4.4	${<}0.1$	0.2–2.7
${\mathcal {S}}$	($e^{+}e^{-}$)	0.6–3.6	0.1–0.3	0.2–2.4	0.1–1.6	0.1–5.0	0.1–0.4	0.2–3.4
	($\mu ^{+}\mu ^{-}$)	0.5–2.9	0.2–0.7	0.2–2.2	0.1–1.1	0.1–4.4	${<}0.1$	0.1–3.1
${\mathcal {F}}$	($e^{+}e^{-}$)	0.6–3.8	0.1–0.4	0.1–2.0	0.1–0.9	0.1–7.4	0.1–0.4	0.2–2.7
	($\mu ^{+}\mu ^{-}$)	0.5–3.0	0.1–0.6	0.1–2.4	0.1–1.3	0.1–1.6	${<}0.1$	0.1–3.2

The total systematic uncertainties were constructed by adding the above systematic uncertainties in quadrature. The systematic uncertainties in the electron channel were typically slightly larger than the ones obtained in the muon channel. They are of the order of 5 to 10 % for those observables where the track reconstruction systematic uncertainties are large ($N_{\mathrm{ch}}$, $\sum p_\text {T}$, beam thrust). For all other observables the systematic uncertainties rarely exceed 5 % and are typically of the order of 2 %. Tables 1, 2, 3 and 4 provide an overview of the range of the relative statistical and systematic uncertainties for all six observables separately for the electron channel and the muon channel in the four $p_\text {T}(\ell ^{+}\ell ^{-})$ ranges. All systematic uncertainties except the lepton-specific uncertainties are highly correlated between the electron channel and the muon channel.

**Table 3:** Ranges of the relative uncertainties $\frac{\delta _{{\mathcal {O}}}}{{\mathcal {O}}}$ of the event-shape observables ${\mathcal {O}}$ for the electron and muon channels indicated by ($e^{+}e^{-}$) and ($\mu ^{+}\mu ^{-}$) for the $p_\text {T}(\ell ^{+}\ell ^{-})$ range 12–25 ${\mathrm{GeV}}$ in percent. The superscripts denote the statistical (‘stat’) and the individual systematic uncertainties in the lepton reconstruction and identification (‘Lepton’), track reconstruction efficiency (‘Tracking’), non-primary particles (‘Non-prim.’), pile-up correction (‘PU’), multijet background (‘Multijet’), and the unfolding (‘Unfold’)

Observable	Channel	${\delta ^{\mathrm{stat}}_{{\mathcal {O}}}}$ $[\%]$	${\delta ^{\mathrm{Lepton}}_{{\mathcal {O}}}}$ $[\%]$	${\delta ^{\mathrm{Tracking}}_{{\mathcal {O}}}}$ $[\%]$	${\delta ^{\mathrm{Non-Prim.}}_{{\mathcal {O}}}}$ $[\%]$	${\delta ^{\mathrm{PU}}_{{\mathcal {O}}}}$ $[\%]$	${\delta ^{\mathrm{Multijet}}_{{\mathcal {O}}}}$ $[\%]$	${\delta ^{\mathrm{Unfold}}_{{\mathcal {O}}}}$ $[\%]$
$N_{\mathrm{ch}}$	($e^{+}e^{-}$)	1–18.8	0.1–2.8	0.24–9.9	0.14–4.5	0.2–22	${<}0.1$–0.5	0.1–4.7
	($\mu ^{+}\mu ^{-}$)	0.8–14.3	0.1–1.9	0.15–9.2	0.2–1.6	0.1–18	${<}0.1$–0.6	${<}0.1$–3.7
$\sum p_{\text {T}}$	($e^{+}e^{-}$)	1.2–4.8	0.1–0.7	0.1–4.3	0.1–1.9	0.5–6	${<}0.1$–0.4	${<}0.1$–1
	($\mu ^{+}\mu ^{-}$)	0.9–3.6	0.1–1.4	0.1–4.6	${<}0.1$–1.8	${<}0.1$–1.4	${<}0.1$–0.3	0.1–2
${\mathcal {B}}$	($e^{+}e^{-}$)	0.8–5.7	0.1–0.8	0.1–3.7	0.1–1.4	0.1–9.1	0.1–1.4	0.1–2.7
	($\mu ^{+}\mu ^{-}$)	0.6–4.3	0.14–1	${<}0.1$–3.9	0.1–0.9	0.18–4.9	${<}0.1$–0.5	${<}0.1$–1.7
${\mathcal {T}}$	($e^{+}e^{-}$)	0.7–5.0	0.1–0.5	0.1–2.8	0.1–1.8	0.1–5.4	0.1–0.8	0.2–3.7
	($\mu ^{+}\mu ^{-}$)	0.5–3.9	0.1–0.5	0.1–2.3	0.1–1.2	0.1–4.9	${<}0.1$	0.2–3.7
${\mathcal {S}}$	($e^{+}e^{-}$)	0.7–3.2	0.1–0.3	0.3–2.4	0.2–1.3	0.1–2.8	0.1–0.9	0.1–4.7
	($\mu ^{+}\mu ^{-}$)	0.5–2.4	0.1–0.4	0.2–2.1	0.1–1.1	0.1–2.5	${<}0.1$	0.1–4.6
${\mathcal {F}}$	($e^{+}e^{-}$)	0.7–3.7	0.1–0.3	0.1–2.2	0.1–1.2	0.2–4.4	0.1–1	0.1–2.1
	($\mu ^{+}\mu ^{-}$)	0.5–2.8	0.1–0.5	0.1–1.7	0.1–1	0.1–2	${<}0.1$	0.1–1.8

**Table 4:** Ranges of the relative uncertainties $\frac{\delta _{{\mathcal {O}}}}{{\mathcal {O}}}$ of the event-shape observables ${\mathcal {O}}$ for the electron and muon channels indicated by ($e^{+}e^{-}$) and ($\mu ^{+}\mu ^{-}$) for $p_\text {T}(\ell ^{+}\ell ^{-}) >25$ ${\mathrm{GeV}}$ in percent. The superscripts denote the statistical (‘stat’) and the individual systematic uncertainties in the lepton reconstruction and identification (‘Lepton’), track reconstruction efficiency (‘Tracking’), non-primary particles (‘Non-prim.’), pile-up correction (‘PU’), multijet background (‘Multijet’), and the unfolding (‘Unfold’)

Observable	Channel	${\delta ^{\mathrm{stat}}_{{\mathcal {O}}}}$ $[\%]$	${\delta ^{\mathrm{Lepton}}_{{\mathcal {O}}}}$ $[\%]$	${\delta ^{\mathrm{Tracking}}_{{\mathcal {O}}}}$ $[\%]$	${\delta ^{\mathrm{Non-Prim.}}_{{\mathcal {O}}}}$ $[\%]$	${\delta ^{\mathrm{PU}}_{{\mathcal {O}}}}$ $[\%]$	${\delta ^{\mathrm{Multijet}}_{{\mathcal {O}}}}$ $[\%]$	${\delta ^{\mathrm{Unfold}}_{{\mathcal {O}}}}$ $[\%]$
$N_{\mathrm{ch}}$	($e^{+}e^{-}$)	1.1–47	0.1–2.5	0.3–8.9	${<}0.1$–15	${<}0.1$–34	0.1–3.5	${<}0.1$–2.1
	($\mu ^{+}\mu ^{-}$)	0.9–28	0.1–3.5	0.2–6.9	${<}0.1$–5.3	0.14–34	${<}0.1$–0.2	0.1–8.9
$\sum p_{\text {T}}$	($e^{+}e^{-}$)	1–8.9	0.1–1.2	0.3–4.1	0.1–1.2	0.1–2.5	${<}0.1$–1.2	0.1–1.4
	($\mu ^{+}\mu ^{-}$)	0.7–6.3	${<}0.1$–1	0.4–4.1	0.1–1.7	${<}0.1$–3.2	${<}0.1$–0.2	0.1–2.1
${\mathcal {B}}$	($e^{+}e^{-}$)	1–3	0.1–0.3	0.2–2.7	0.2–0.7	0.1–2.3	0.1–0.9	0.1–1.7
	($\mu ^{+}\mu ^{-}$)	0.8–2.2	0.1–0.6	0.3–2.9	0.1–0.8	0.1–1.5	${<}0.1$–0.1	${<}0.1$–1.6
${\mathcal {T}}$	($e^{+}e^{-}$)	0.9–4.4	0.1–0.3	0.1–1.5	0.1–1.8	0.1–5.3	0.1–0.8	0.4–3.7
	($\mu ^{+}\mu ^{-}$)	0.7–3.5	0.1–0.5	0.1–1.6	0.1–1.9	0.1–3.7	${<}0.1$	0.1–4.3
${\mathcal {S}}$	($e^{+}e^{-}$)	0.9–3.9	0.1–0.6	0.1–1.8	0.1–1.1	0.2–12.3	0.1–0.8	0.1–2.7
	($\mu ^{+}\mu ^{-}$)	0.7–3.1	0.1–0.7	0.1–1.8	0.1–0.5	0.1–8	${<}0.1$	0.1–6.2
${\mathcal {F}}$	($e^{+}e^{-}$)	0.9–2.8	0.1–0.3	0.1–0.8	0.1–1.1	0.1–5.4	0.1–0.8	0.1–2.4
	($\mu ^{+}\mu ^{-}$)	0.7–2.1	0.1–0.6	0.1–0.9	0.1–0.9	0.1–2.7	${<}0.1$	0.1–0.9

## Results

The results from the electron and muon channels are in good agreement and numerical values for each channel are provided in HEPDATA [55]. The statistical uncertainties in the muon results are slightly smaller than those in the electron results and in general the results are dominated by the systematic uncertainties. Since the electron- and muon-specific systematic uncertainties are smaller than the common dominant systematic uncertainties in the track reconstruction efficiency, the PU correction factors, and the unfolding model, the electron and muon results were not combined.

**Fig. 1:**
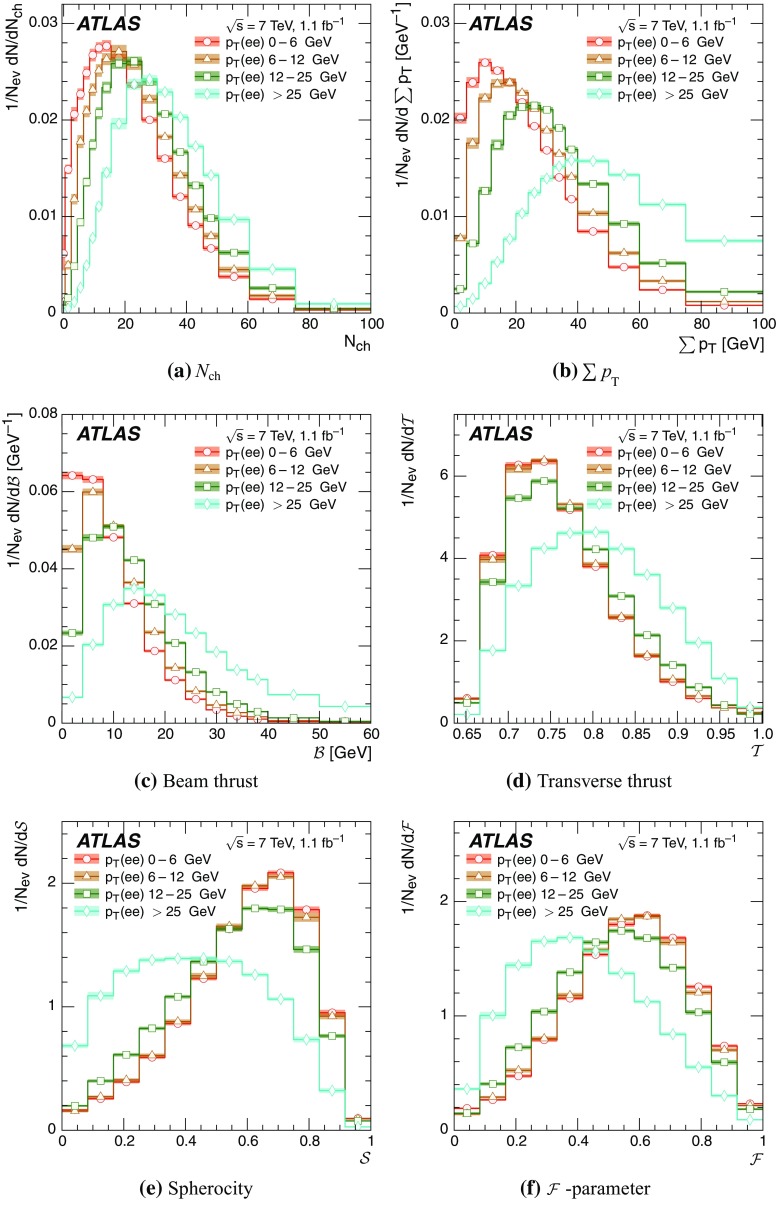
Distributions of the event-shape variables **a** charged-particle multiplicity $N_{\mathrm{ch}}$, **b** summed transverse momenta $\sum p_{\text {T}}$, **c** beam thrust ${\mathcal {B}}$, **d** transverse thrust ${\mathcal {T}}$, **e** spherocity ${\mathcal {S}}$, and **f** ${\mathcal {F}}$-parameter as defined in Sect. 2 measured in $Z \rightarrow e^{+} e^{-}$ events for the different ranges of the transverse momentum of the $e^{+} e^{-}$ system, $p_\text {T}(e^{+} e^{-})$ (*open circles* 0–6 ${\mathrm{GeV}}$, *open triangles* 6–12 ${\mathrm{GeV}}$, *open boxes* 12–25 ${\mathrm{GeV}}$, *open diamonds* ${\ge } 25$ ${\mathrm{GeV}}$). $N_{\text {ev}}$ denotes the number of events in the $p_\text {T}(e^{+} e^{-})$ range passing the analysis cuts. The *bands* show the sum in quadrature of the statistical and all systematic uncertainties

**Fig. 2:**
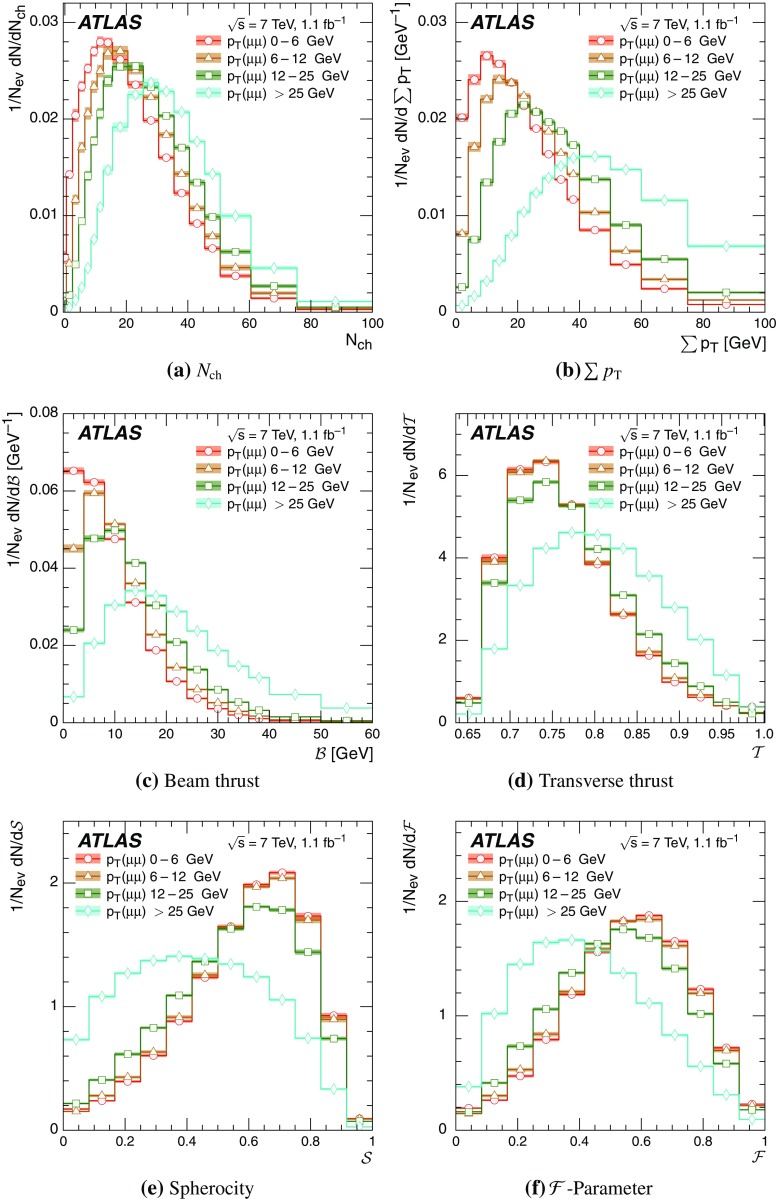
Distributions of the event-shape variables **a** charged-particle multiplicity $N_{\mathrm{ch}}$, **b** summed transverse momenta $\sum p_{\text {T}}$, **c** beam thrust ${\mathcal {B}}$, **d** transverse thrust ${\mathcal {T}}$, **e** spherocity ${\mathcal {S}}$, and **f** ${\mathcal {F}}$-parameter as defined in Sect. 2 measured in $Z \rightarrow \mu ^{+} \mu ^{-}$ events for the different ranges of the transverse momentum of the $\mu ^{+} \mu ^{-}$ system, $p_\text {T}(\mu ^{+} \mu ^{-})$ (*open circles* 0–6 ${\mathrm{GeV}}$, *open triangles* 6–12 ${\mathrm{GeV}}$, *open boxes* 12–25 ${\mathrm{GeV}}$, *open diamonds* ${\ge } 25$ ${\mathrm{GeV}}$). $N_{\text {ev}}$ denotes the number of events in the $p_\text {T}(\mu ^{+} \mu ^{-})$ range passing the analysis cuts. The *bands* show the sum in quadrature of the statistical and all systematic uncertainties

Figure 1 (Fig. 2) shows the unfolded electron (muon) channel results for the six observables in the various $p_\text {T}(\ell ^{+}\ell ^{-})$ ranges, with the total uncertainty presented as the quadratic sum of the statistical and total systematic uncertainties. As $p_\text {T}(\ell ^{+}\ell ^{-})$ rises, i.e. as recoiling jets emerge, the number of produced charged particles $N_{\mathrm{ch}}$ increases, as do $\sum p_{\text {T}}$ and beam thrust. Correspondingly, transverse thrust moves towards higher values and spherocity towards smaller values as a result of the increasing jettiness of the events.

**Fig. 3:**
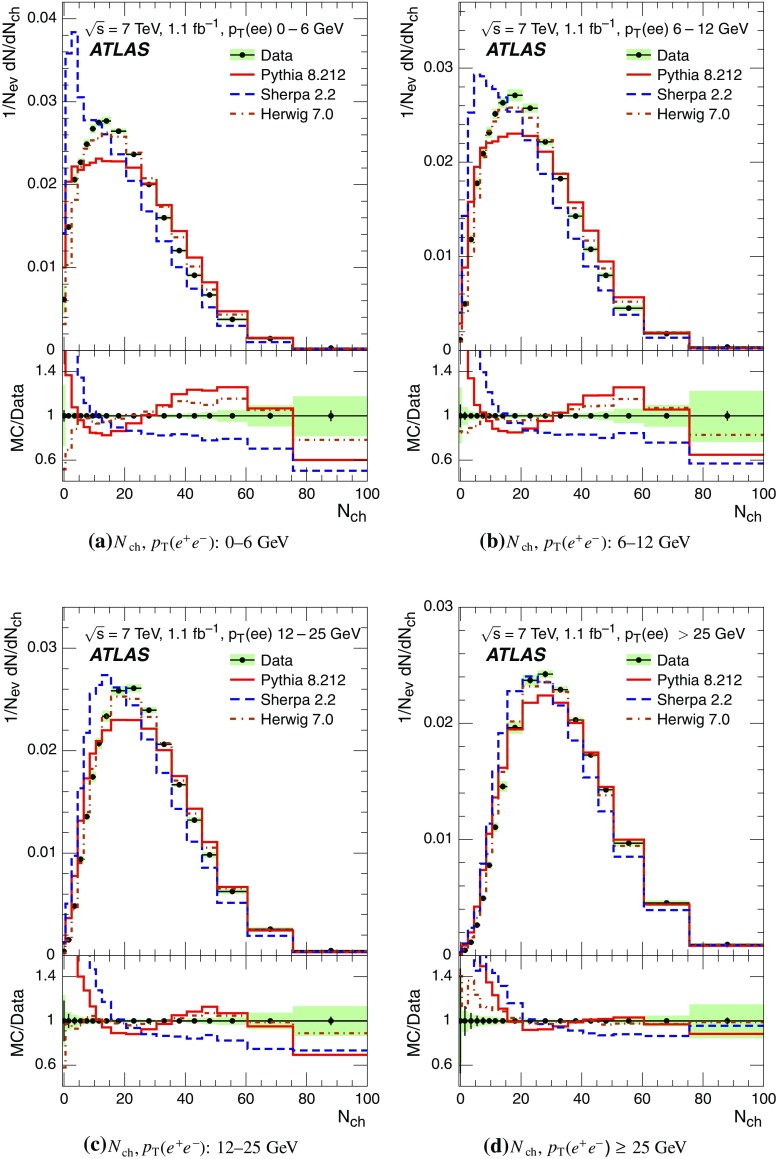
Distribution of charged-particle multiplicity, $N_{\mathrm{ch}}$, for $Z \rightarrow e^{+}e^{-}$ with statistical (*error bars*) and total systematic (*band*) uncertainties for the four $p_\text {T}(e^{+} e^{-})$ ranges (**a** 0–6 ${\mathrm{GeV}}$, **b** 6–12 ${\mathrm{GeV}}$, **c** 12–25 ${\mathrm{GeV}}$, **d** ${\ge } 25$ ${\mathrm{GeV}}$) compared to the predictions from the MC generators Pythia 8 (*full line*), Sherpa (*dashed line*), and Herwig 7 (*dashed-dotted line*). In each subfigure, the *top plot* shows the observable and the *bottom plot* shows the ratio of the MC simulation to the data

**Fig. 4:**
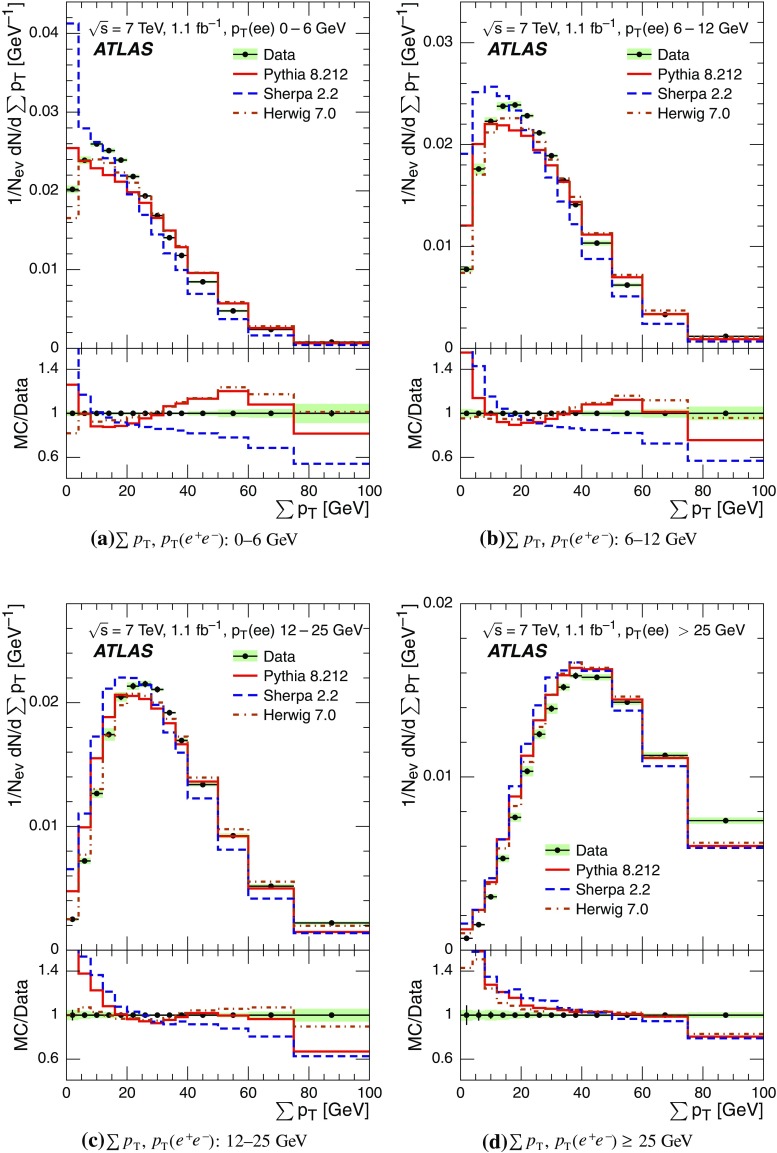
Summed transverse momenta $\sum p_{\text {T}}$ distribution of charged particles for $Z \rightarrow e^{+}e^{-}$ with statistical (*error bars*) and total systematic (*band*) uncertainties for the four $p_\text {T}(e^{+} e^{-})$ ranges (**a** 0–6 ${\mathrm{GeV}}$, **b** 6–12 ${\mathrm{GeV}}$, **c** 12–25 ${\mathrm{GeV}}$, **d** ${\ge } 25$ ${\mathrm{GeV}}$) compared to the predictions from the MC generators Pythia 8 (*full line*), Sherpa (*dashed line*), and Herwig 7 (*dashed-dotted line*). In each subfigure, the *top plot* shows the observable and the *bottom plot* shows the ratio of the MC simulation to the data

**Fig. 5:**
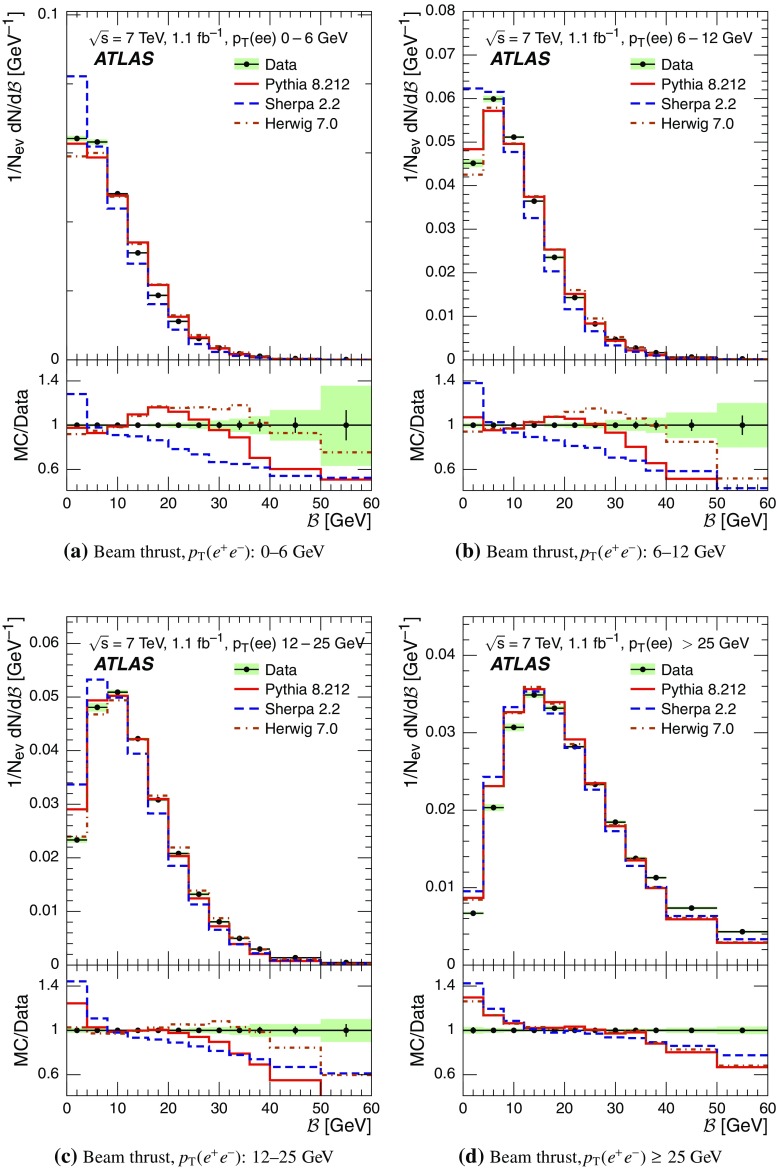
Beam thrust ${\mathcal {B}}$ distribution of charged particles for $Z \rightarrow e^{+}e^{-}$ with statistical (*error bars*) and total systematic (*band*) uncertainties for the four $p_\text {T}(e^{+} e^{-})$ ranges (**a** 0–6 ${\mathrm{GeV}}$, **b** 6–12 ${\mathrm{GeV}}$, **c** 12–25 ${\mathrm{GeV}}$, **d** ${\ge } 25$ ${\mathrm{GeV}}$) compared to the predictions from the MC generators Pythia 8 (*full line*), Sherpa (*dashed line*), and Herwig 7 (*dashed-dotted line*). In each subfigure, the *top plot* shows the observable and the *bottom plot* shows the ratio of the MC simulation to the data

**Fig. 6:**
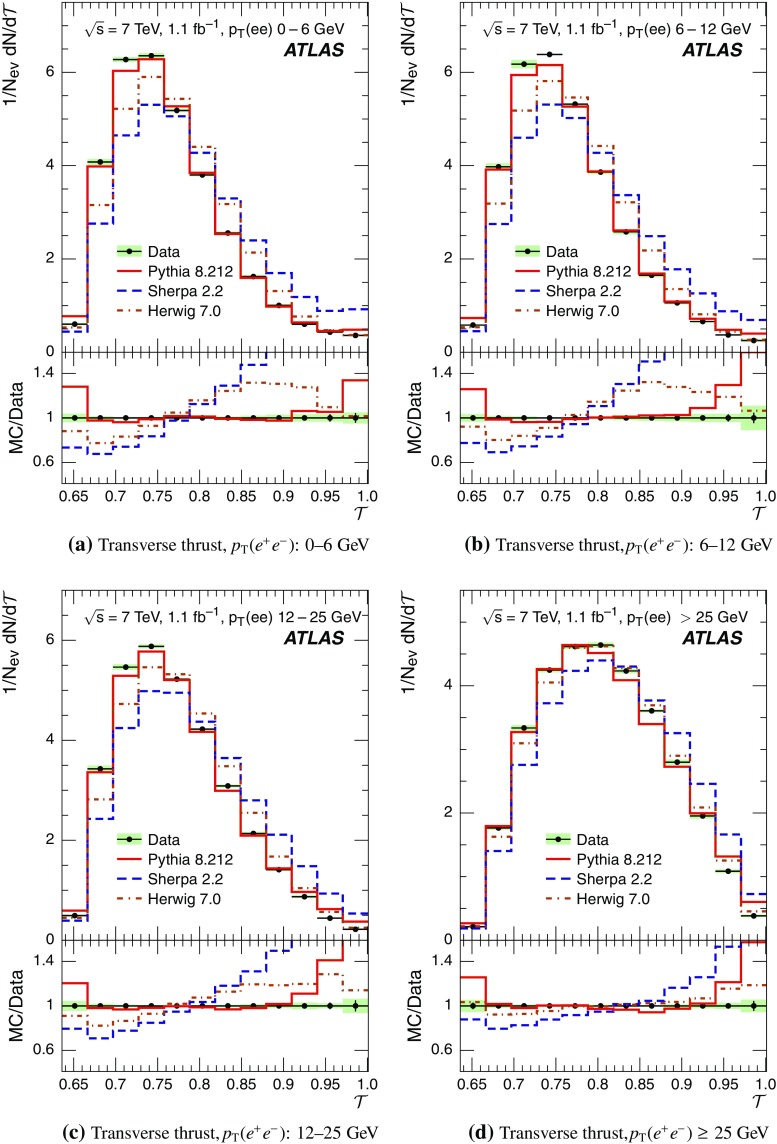
Transverse thrust ${\mathcal {T}}$ distribution of charged particles for $Z \rightarrow e^{+}e^{-}$ with statistical (*error bars*) and total systematic (*band*) uncertainties for the four $p_\text {T}(e^{+} e^{-})$ ranges (**a** 0–6 ${\mathrm{GeV}}$, **b** 6–12 ${\mathrm{GeV}}$, **c** 12–25 ${\mathrm{GeV}}$, **d** ${\ge } 25$ ${\mathrm{GeV}}$) compared to the predictions from the MC generators Pythia 8 (*full line*), Sherpa (*dashed line*), and Herwig 7 (*dashed-dotted line*). In each subfigure, the *top plot* shows the observable and the *bottom plot* shows the ratio of the MC simulation to the data

**Fig. 7:**
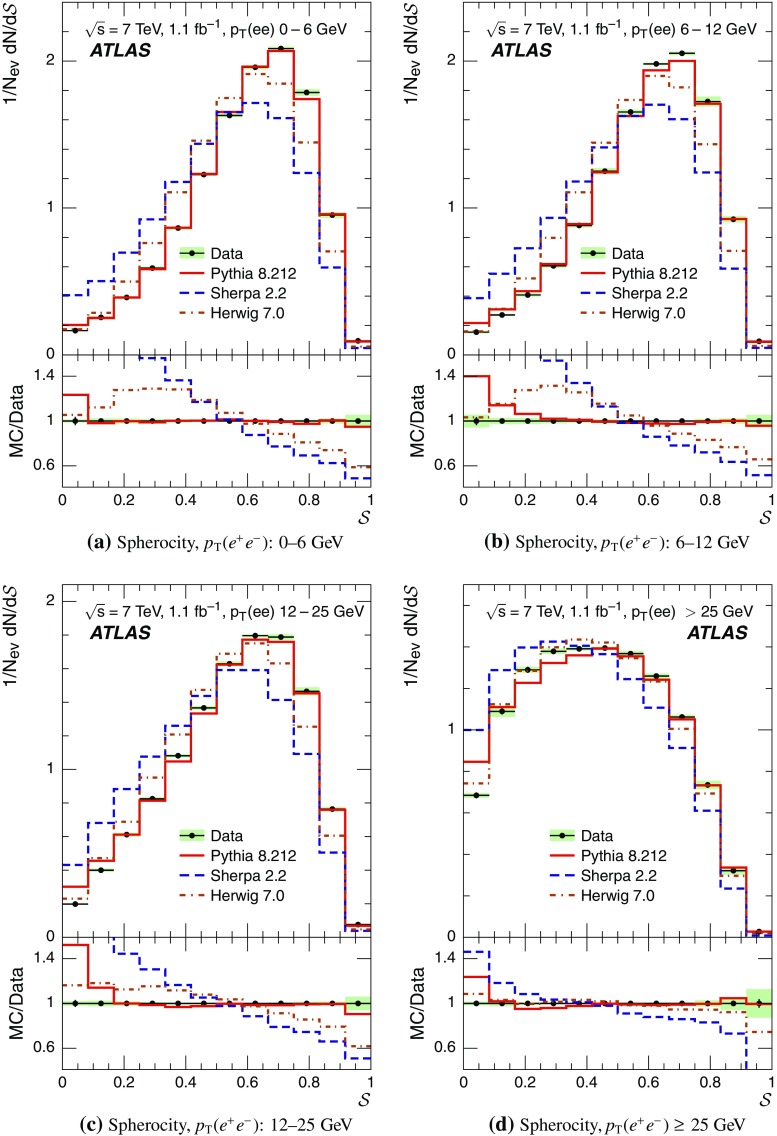
Spherocity ${\mathcal {S}}$ distribution of charged particles for $Z \rightarrow e^{+}e^{-}$ with statistical (*error bars*) and total systematic (*band*) uncertainties for the four $p_\text {T}(e^{+} e^{-})$ ranges (**a** 0–6 ${\mathrm{GeV}}$, **b** 6–12 ${\mathrm{GeV}}$, **c** 12–25 ${\mathrm{GeV}}$, **d** ${\ge } 25$ ${\mathrm{GeV}}$) compared to the predictions from the MC generators Pythia 8 (*full line*), Sherpa (*dashed line*), and Herwig 7 (*dashed-dotted line*). In each subfigure, the *top plot* shows the observable and the *bottom plot* shows the ratio of the MC simulation to the data

**Fig. 8:**
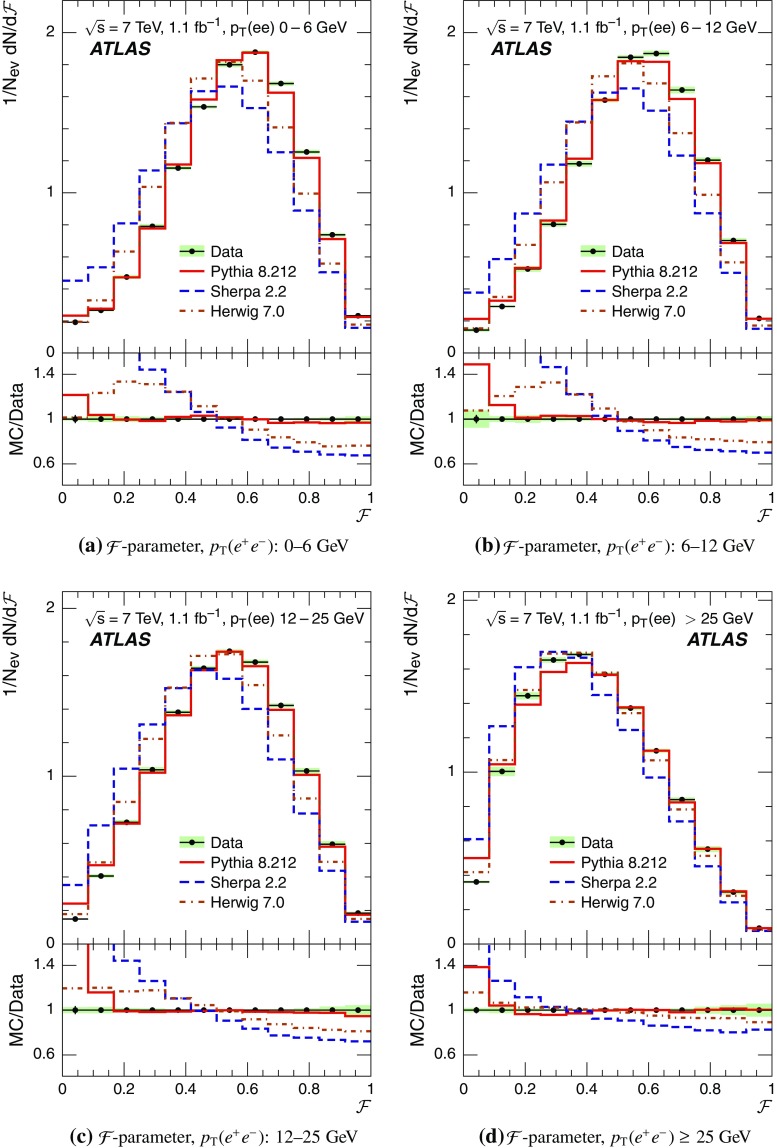
${\mathcal {F}}$-parameter distribution of charged particles for $Z \rightarrow e^{+}e^{-}$ with statistical (*error bars*) and total systematic (*band*) uncertainties for the four $p_\text {T}(e^{+} e^{-})$ ranges (**a** 0–6 ${\mathrm{GeV}}$, **b** 6–12 ${\mathrm{GeV}}$, **c** 12–25 ${\mathrm{GeV}}$, **d** ${\ge } 25$ ${\mathrm{GeV}}$) compared to the predictions from the MC generators Pythia 8 (*full line*), Sherpa (*dashed line*), and Herwig 7 (*dashed-dotted line*). In each subfigure, the top plot shows the observable and the bottom plot shows the ratio of the MC simulation to the data

**Fig. 9:**
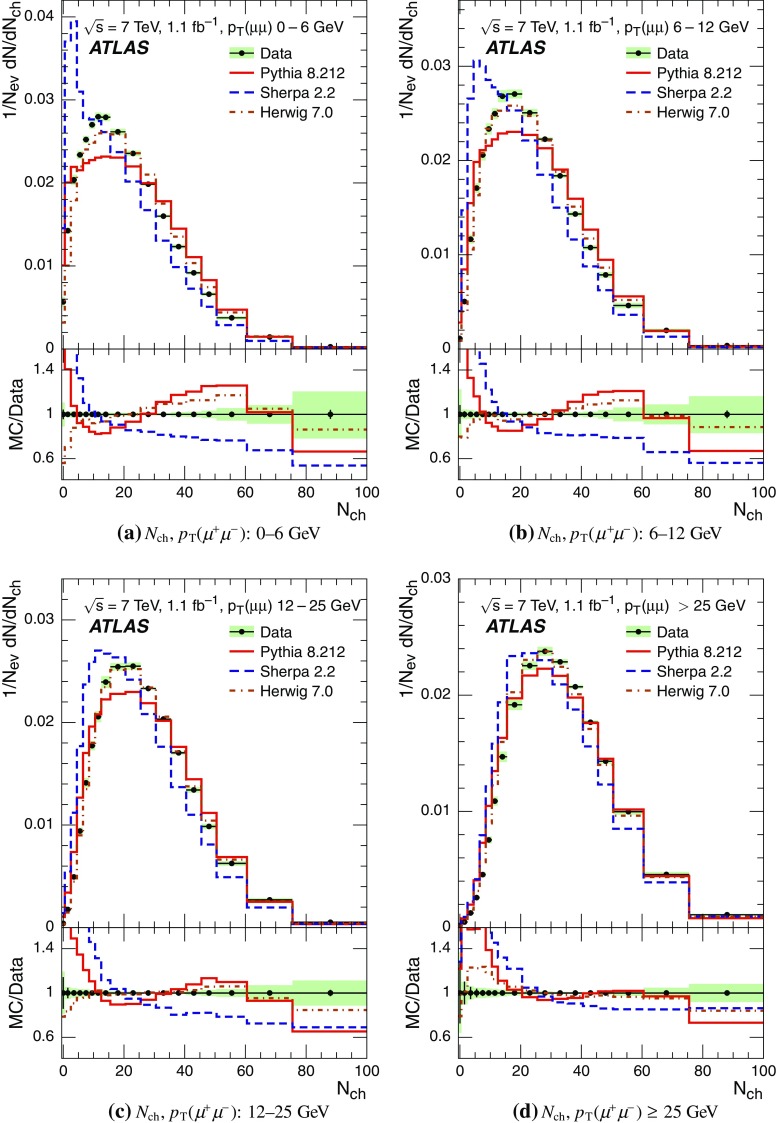
Distribution of charged-particle multiplicity, $N_{\mathrm{ch}}$, for $Z \rightarrow \mu ^{+}\mu ^{-}$ with statistical (*error bars*) and total systematic (*band*) uncertainties for the four $p_\text {T}(\mu ^{+} \mu ^{-})$ ranges (**a** 0–6 ${\mathrm{GeV}}$, **b** 6–12 ${\mathrm{GeV}}$, **c** 12–25 ${\mathrm{GeV}}$, **d** ${\ge } 25$ ${\mathrm{GeV}}$) compared to the predictions from the MC generators Pythia 8 (*full line*), Sherpa (*dashed line*), and Herwig 7 (*dashed-dotted line*). In each subfigure, the *top plot* shows the observable and the *bottom plot* shows the ratio of the MC simulation to the data

**Fig. 10:**
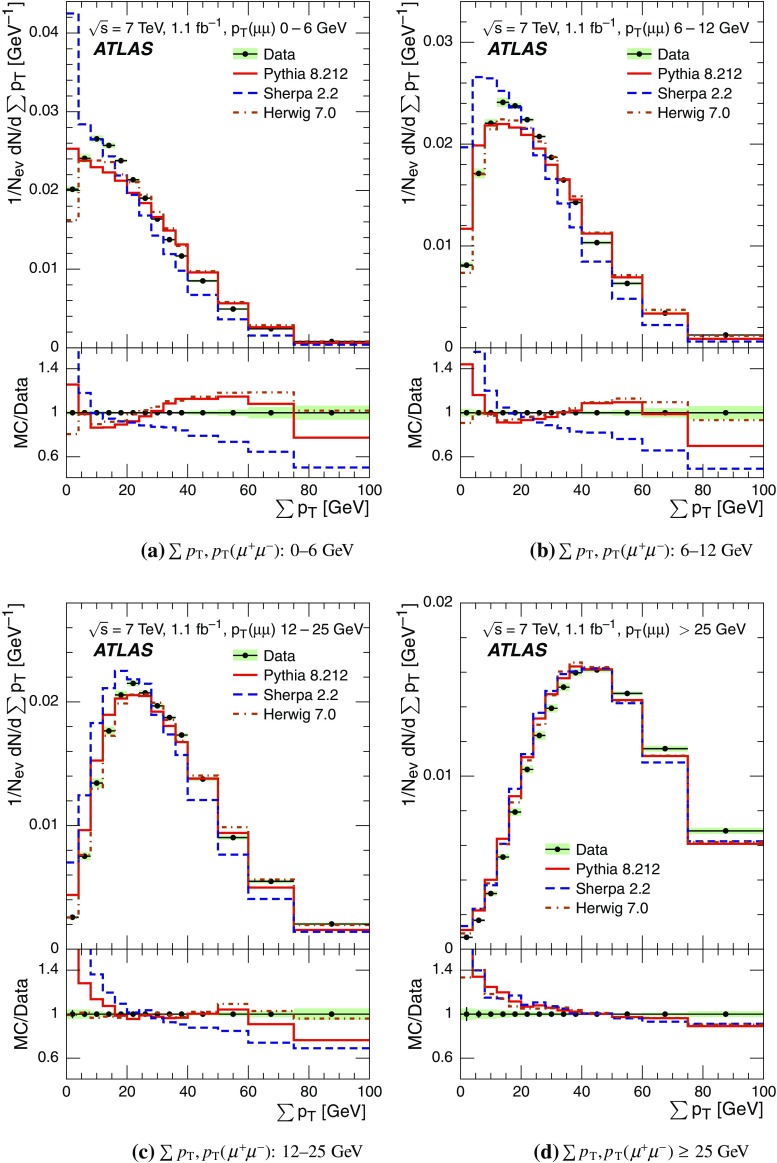
Summed transverse momenta $\sum p_{\text {T}}$ distribution of charged particles for $Z \rightarrow \mu ^{+}\mu ^{-}$ with statistical (*error bars*) and total systematic (*band*) uncertainties for the four $p_\text {T}(\mu ^{+} \mu ^{-})$ ranges (**a** 0–6 ${\mathrm{GeV}}$, **b** 6–12 ${\mathrm{GeV}}$, **c** 12–25 ${\mathrm{GeV}}$, **d** ${\ge } 25$ ${\mathrm{GeV}}$) compared to the predictions from the MC generators Pythia 8 (*full line*), Sherpa (*dashed line*), and Herwig 7 (*dashed-dotted line*). In each subfigure, the *top plot* shows the observable and the *bottom plot* shows the ratio of the MC simulation to the data

**Fig. 11:**
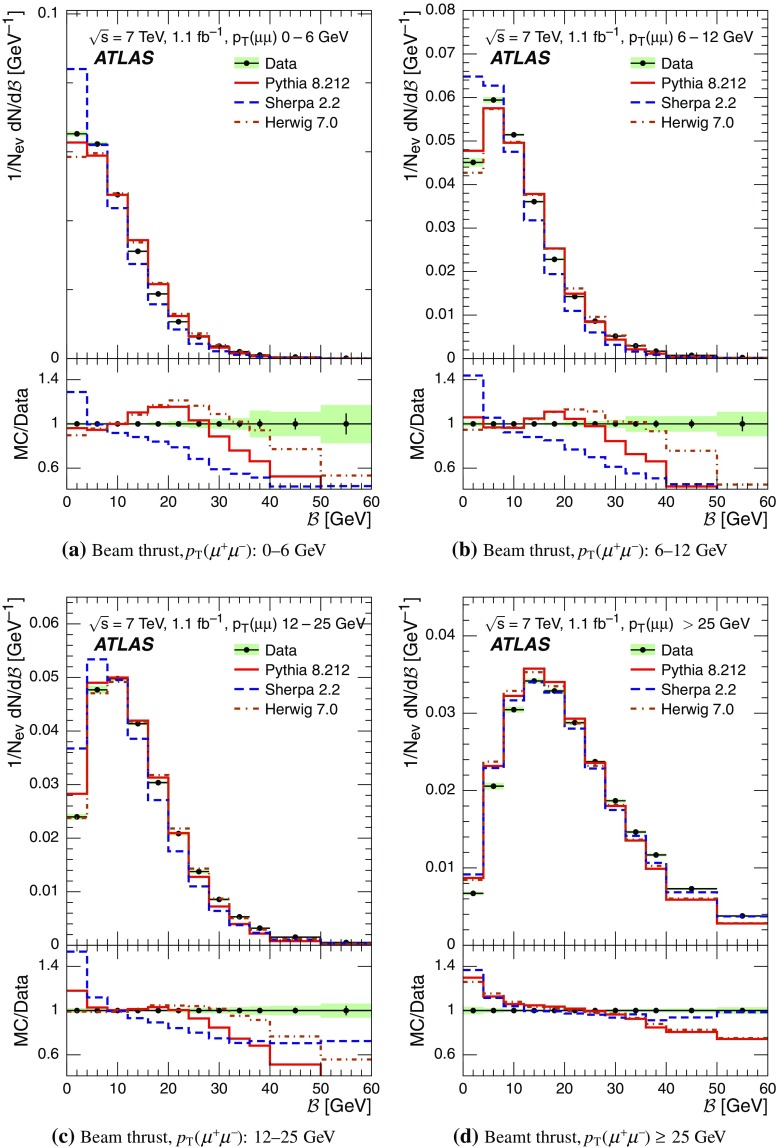
Beam thrust ${\mathcal {B}}$ distribution of charged particles for $Z \rightarrow \mu ^{+}\mu ^{-}$ with statistical (*error bars*) and total systematic (*band*) uncertainties for the four $p_\text {T}(\mu ^{+} \mu ^{-})$ ranges (**a** 0–6 ${\mathrm{GeV}}$, **b** 6–12 ${\mathrm{GeV}}$, **c** 12–25 ${\mathrm{GeV}}$, **d** ${\ge } 25$ ${\mathrm{GeV}}$) compared to the predictions from the MC generators Pythia 8 (*full line*), Sherpa (*dashed line*), and Herwig 7 (*dashed-dotted line*). In each subfigure, the *top plot* shows the observable and the *bottom plot* shows the ratio of the MC simulation to the data

**Fig. 12:**
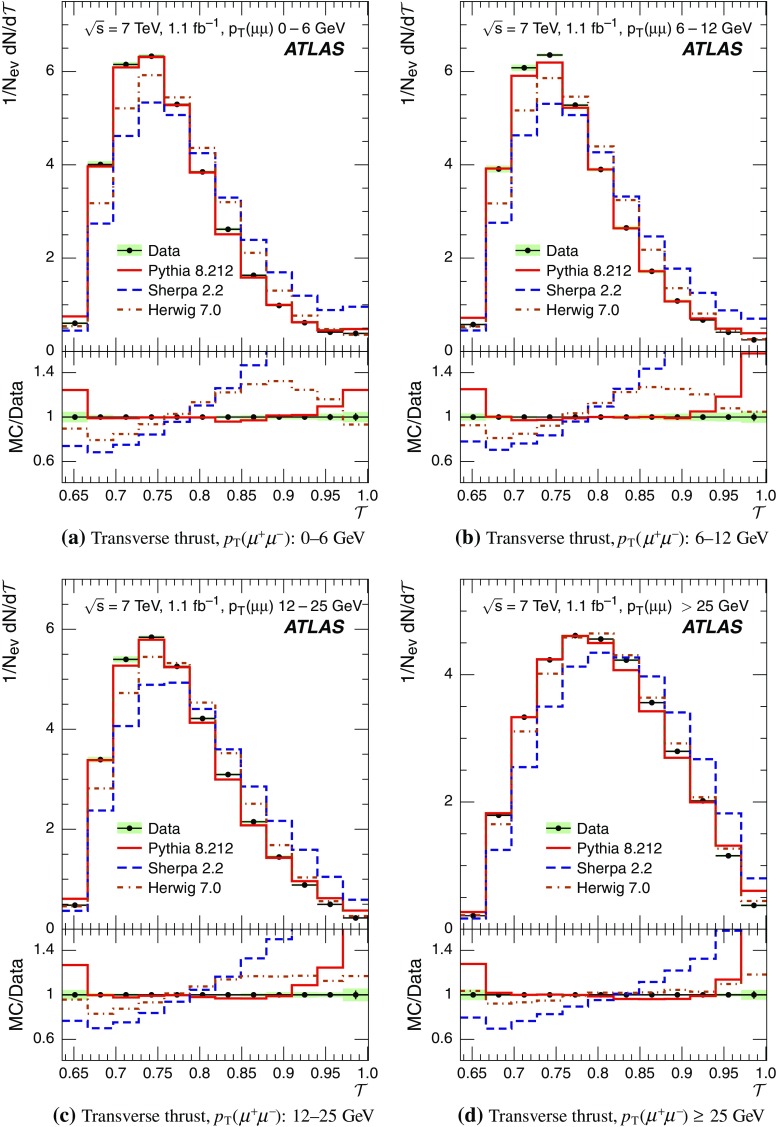
Transverse thrust ${\mathcal {T}}$ distribution of charged particles for $Z \rightarrow \mu ^{+}\mu ^{-}$ with statistical (*error bars*) and total systematic (*band*) uncertainties for the four $p_\text {T}(\mu ^{+} \mu ^{-})$ ranges (**a** 0–6 ${\mathrm{GeV}}$, **b** 6–12 ${\mathrm{GeV}}$, **c** 12–25 ${\mathrm{GeV}}$, **d** ${\ge } 25$ ${\mathrm{GeV}}$) compared to the predictions from the MC generators Pythia 8 (*full line*), Sherpa (*dashed line*), and Herwig 7 (*dashed-dotted line*). In each subfigure, the *top plot* shows the observable and the *bottom plot* shows the ratio of the MC simulation to the data

**Fig. 13:**
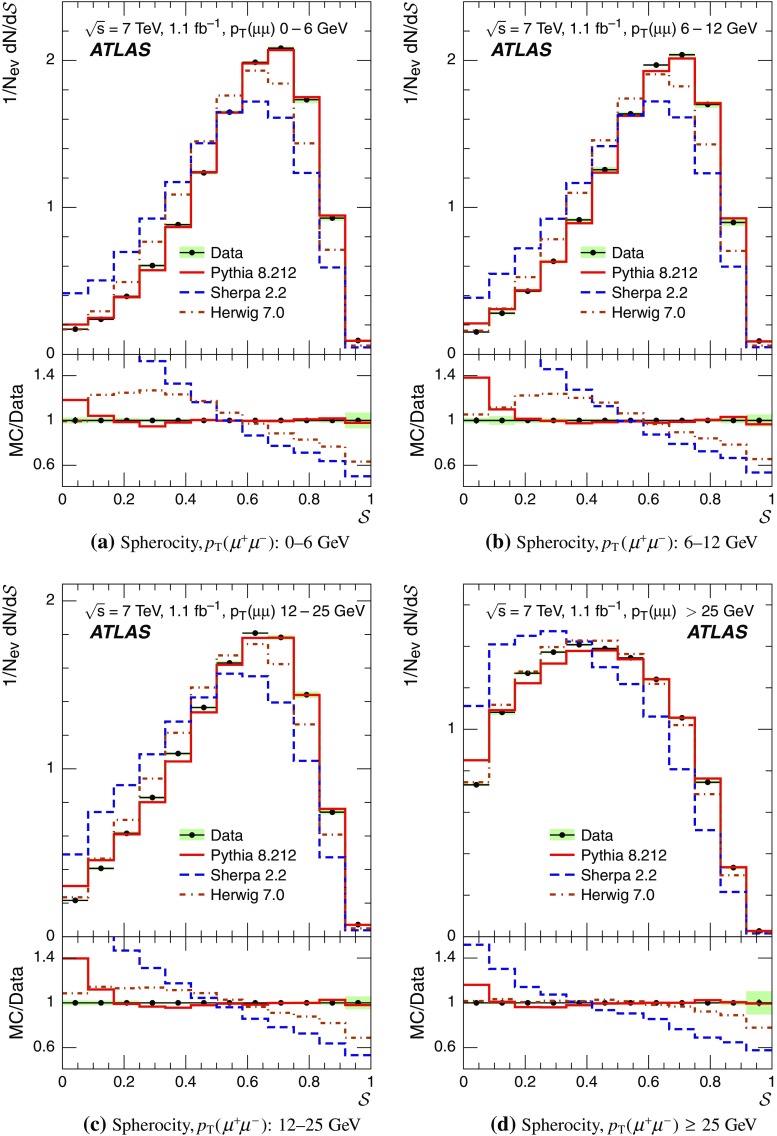
Spherocity ${\mathcal {S}}$ distribution of charged particles for $Z \rightarrow \mu ^{+}\mu ^{-}$ with statistical (*error bars*) and total systematic (*band*) uncertainties for the four $p_\text {T}(\mu ^{+} \mu ^{-})$ ranges (**a** 0–6 ${\mathrm{GeV}}$, **b** 6–12 ${\mathrm{GeV}}$, **c** 12–25 ${\mathrm{GeV}}$, **d** ${\ge } 25$ ${\mathrm{GeV}}$) compared to the predictions from the MC generators Pythia 8 (*full line*), Sherpa (*dashed line*), and Herwig 7 (*dashed-dotted line*). In each subfigure, the *top plot* shows the observable and the *bottom plot* shows the ratio of the MC simulation to the data

**Fig. 14:**
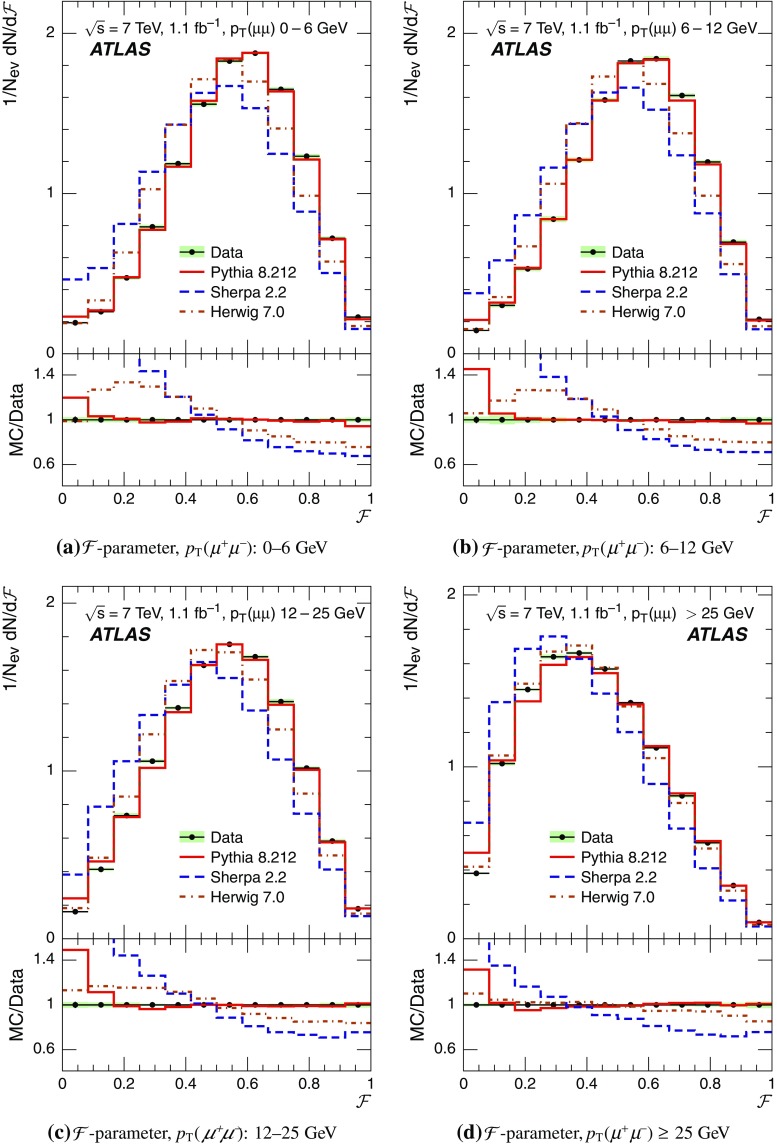
${\mathcal {F}}$-parameter distribution of charged particles for $Z \rightarrow \mu ^{+}\mu ^{-}$ with statistical (*error bars*) and total systematic (*band*) uncertainties for the four $p_\text {T}(\mu ^{+} \mu ^{-})$ ranges (**a** 0–6 ${\mathrm{GeV}}$, **b** 6–12 ${\mathrm{GeV}}$, **c** 12–25 ${\mathrm{GeV}}$, **d** ${\ge } 25$ ${\mathrm{GeV}}$) compared to the predictions from the MC generators Pythia 8 (*full line*), Sherpa (*dashed line*), and Herwig 7 (*dashed-dotted line*). In each subfigure, the *top plot* shows the observable and the *bottom plot* shows the ratio of the MC simulation to the data

Figures 3, 4, 5, 6, 7 and 8 (Figs. 9, 10, 11, 12, 13, 14) show the individual event-shape observables for the electron (muon) channel compared to predictions obtained with the most recent versions of three different MC generators as described in Sect. 4: Sherpa version 2.2.0, Herwig 7 version 7.0, and Pythia 8 version 8.212. In general, Pythia 8 and Herwig 7 agree better with the data than does Sherpa.

The $p_\text {T}(\ell ^{+}\ell ^{-}) <6$ ${\mathrm{GeV}}$ bin is expected to be characterised by low jet activity from the hard matrix element and hence should be particularly sensitive to UE characteristics. In this case, Pythia 8 shows very good agreement with the data in the event-shape observables that are not very sensitive to the number of charged particles (${\mathcal {T}}$, ${\mathcal {S}}$, and ${\mathcal {F}}$-parameter). The observables that depend explicitly on the number of charged particles ($N_{\mathrm{ch}}$, $\sum p_{\text {T}}$, ${\mathcal {B}}$) are less well described, with none of the generators succeeding fully. In this case, the best agreement is observed for Herwig 7 while Pythia 8 still performs better than Sherpa. Low $N_{\mathrm{ch}}$ and $\sum p_{\text {T}}$ values represent a challenging region for all three generators: while Pythia 8 and Sherpa overestimate the data, Herwig 7 significantly underestimates the measurements. This region might be particularly sensitive to the way beam-remnant interactions are modelled in the MC generators. Similar observations can be made for $p_\text {T}(\ell ^{+}\ell ^{-})$ ranges 6–12 and 12–25 ${\mathrm{GeV}}$. At low values of ${{\mathcal {B}}}$, the observable in which tracks with larger $|\eta ^\text {trk}|$ values contribute less to the sum of the track transverse momenta, better agreement of the generator predictions with the data is observed than at low $\sum p_{\text {T}}$.

At $p_\text {T}(\ell ^{+}\ell ^{-}) \ge 25$ ${\mathrm{GeV}}$ the event is expected to contain at least one jet of high transverse momentum recoiling against the *Z* boson, which is expected to be well described by the hard matrix element. In this case, one still observes significant deviations of the MC generators from the measurement, where, depending on the observable, either Herwig 7 or Pythia 8 shows in general the best agreement. However, all three generators show better agreement with data compared to the $p_\text {T}(\ell ^{+}\ell ^{-}) <6$ ${\mathrm{GeV}}$ range.

The observed deviations of MC predictions from the measured observables reveal that MC parameters tuned to presently measured observables fail to describe more detailed characteristics of the UE modelling and the level of disagreement depends on the generator under consideration. It has to be seen whether these discrepancies can be reduced by a refined parameter tuning when also including the event-shape observables in the tuning or whether further developments in the UE modelling are required.

## Conclusion

In this paper, event-shape observables sensitive to the underlying event were measured in 1.1 $\mathrm{fb}^{-1}$ integrated luminosity of proton–proton collisions collected with the ATLAS detector at the LHC at a centre-of-mass energy of 7 TeV. Events containing an oppositely charged electron or muon pair with an invariant mass close to the *Z*-boson mass were selected, and the charged particle multiplicity, mean transverse momentum, beam thrust, transverse thrust, spherocity, and ${\mathcal {F}}$-parameter were measured, excluding the particles from the *Z*-boson decay.

The measured observables were corrected for the effect of pile-up and multijet background, and then for contributions from non-primary particles, detector efficiency, and resolution effects using an unfolding technique. The resulting distributions are presented in different regions of the *Z*-boson transverse momentum and compared to predictions of the MC event generators Pythia 8, Herwig 7 and Sherpa. These comparisons reveal significant deviations of the Sherpa predictions from the measured observables. Depending on the observable under consideration and the transverse momentum of the *Z* boson, the data are in much better agreement with the Pythia 8 and Herwig 7 predictions than with Sherpa.

Typically, all three Monte Carlo generators provide predictions that are in better agreement with the data at high *Z*-boson transverse momenta than at low *Z*-boson transverse momenta and for the observables that are less sensitive to the number of charged particles in the event (transverse thrust, spherocity, and ${\mathcal {F}}$-parameter). The Monte Carlo generator predictions show significant differences from the data at low values of $N_{\mathrm{ch}}$, $\sum p_{\text {T}}$, and beam thrust in certain regions of the *Z*-boson transverse momentum. The measured event-shape observables are therefore expected to provide valuable insight into the phenomenon of the underlying event and new information for the tuning of current underlying-event models and the development of new models for high-precision measurements to be performed at the LHC at $\sqrt{s}=13$ TeV.

## Footnotes

1 The ATLAS reference system is a Cartesian right-handed coordinate system, with the nominal collision point at the origin. The anticlockwise beam direction defines the positive *z*-axis, while the positive *x*-axis is defined as pointing from the collision point to the centre of the LHC ring and the positive *y*-axis points upwards. The azimuthal angle $\phi$ is measured around the beam axis, and the polar angle $\theta$ is measured with respect to the *z*-axis. The pseudorapidity is given by $\eta = -\ln {\tan (\theta /2)}$ and the $\eta$–$\phi$ distance between two objects *i* and *j* by ${\Delta R_{i,j}} = \sqrt{(\eta _{i}-\eta _{j})^2 + (\phi _{i}-\phi _{j})^2}$. The transverse momentum $p_\text {T}$ is defined relative to the beam axis.

2 It is implicitly understood that the lepton–antilepton pair is produced from a *Z* boson or virtual photon $\gamma ^{*}$.

3 If not stated explicitly, “electrons” and “muons” denote both the corresponding lepton and antilepton.
